# Geographic Genetic Structure of *Alectoris chukar* in Türkiye: Post-LGM-Induced Hybridization and Human-Mediated Contaminations

**DOI:** 10.3390/biology12030401

**Published:** 2023-03-03

**Authors:** Sarp Kaya, Bekir Kabasakal, Ali Erdoğan

**Affiliations:** 1 First and Emergency Aid Programme, Department of Medical Services and Techniques, Vocational School of Burdur Health Services, Burdur Mehmet Akif Ersoy University, Burdur 15030, Turkey; 2 Department of Biology, Akdeniz University, Antalya 07058, Turkey; 3 Anesthesia Programme, Department of Medical Services and Techniques, Vocational School of Health Services, Antalya Bilim University, Antalya 07190, Turkey

**Keywords:** *Alectoris chukar*, Anatolia, hybrid zone, genetic contaminations, post-LGM, conservation genetics, Turkey

## Abstract

**Simple Summary:**

The chukar partridge (*Alectoris chukar*) is an important bird species with significant economic and ecological value. Every year, thousands of partridges raised in Ministry of Agriculture and Forestry breeding farms are released into nature for hunting purposes. Our investigation of the gene pools of different *A. chukar* populations using mitochondrial and microsatellite markers from across Türkiye revealed that the Eastern, Western, and Thracian genetic structures have been shaped by Türkiye’s geography and topography, past climatic fluctuations, and human-mediated artificial hybridizations. There were no signs of congeneric genetic contamination in native chukar populations, but there were contaminations with the China clade-B, which were detected as reported in the previous studies.

**Abstract:**

Türkiye is considered an important evolutionary area for Chukar partridge (*Alectoris chukar*), since it is both a potential ancestral area and a diversification center for the species. Using 2 mitochondrial (Cty-b and D-loop) and 13 polymorphic microsatellite markers, we investigated the geographic genetic structure of *A. chukar* populations to determine how past climatic fluctuations and human activities have shaped the gene pool of this species in Türkiye. Our results indicate, firstly, that only *A. chukar* of the genus *Alectoris* is present in Türkiye (Anatolia and Thrace), with no natural or artificial gene flow from congenerics. Secondly, the geographic genetic structure of the species in Türkiye has been shaped by topographic heterogeneity, Pleistocene climatic fluctuations, and artificial transport by humans. Third, there appears to be three genetic clusters: Thracian, Eastern, and Western. Fourth, the post-LGM demographic expansion of the Eastern and Western populations has formed a hybrid zone in Central Anatolia (~8 kyBP). Fifth, the rate of China clade-B contamination in Türkiye is about 8% in mtDNA and about 12% in nuDNA, with the Southeastern Anatolian population having the highest contamination. Sixth, the Thracian population was the most genetically distinct, with the lowest genetic diversity and highest level of inbreeding and no China clad-B contamination. These results can contribute to the conservation regarding *A. chukar* populations, especially the Thracian population.

## 1. Introduction

Quaternary climate fluctuations are an important evolutionary mechanism that have shaped the present population structure and species/lineage diversity [1,2,3,4]. During this period, which covers the last 2.58 million years, cosmic forces (Milankovitch cycles) have caused global temperature fluctuations that have led to dramatic climate changes in the Earth’s surface [5]. These fluctuations have, in turn, driven horizontal and vertical migrations of populations [6,7]. Past climatic fluctuations, together with the topographic heterogeneity of particular areas, have caused the differentiation, mixing, and extinction of lineages through spatial and demographic expansions and contractions [8,9,10,11]. More specifically, intense glaciation periods (115–12 kyBP), particularly the Last Glacial Maximum, or LGM (~22 kyBP) [12,13], have significantly affected the distribution and genetic structure of populations, especially in the Northern Hemisphere [6,14,15]. This cooling period forced populations to migrate to refugial areas, which caused fragmentation and shrinkage of populations. 

The Mediterranean Basin is considered an important refugial area for many Western Palearctic lineages [14,16]. Five main peninsulas (Maghreb, Iberia, Italy, the Balkans, and Anatolia) have played important roles in both the preservation and diversification of lineages during Quaternary climate fluctuations, particularly in cooling periods of glaciation [17,18,19,20,21]. In contrast, the retreat of glacial and permafrost layers in Europe’s high latitudes during the warm period allowed faunal elements to re-radiate from these southern refugia into Northern Europe [20,22]. The postglacial recolonization of closely related lineages from different refugia in Europe’s northern latitudes has resulted in the formation of hybrid areas outside the refugia [23,24,25,26,27]. Central and Northern Europe, in particular, are well-known areas where lineages mixed in the post-LGM period (the last 11.6 kyBP) [19,25,28,29]. In the post-LGM period, some lineages from Iberia, Italy, and the Balkan peninsulas have formed contact zones in Central and Northern Europe, while Anatolian lineages and their European relatives have formed contact zones in the Eastern Mediterranean, generally in Thrace and the Balkans [27,29]. These hybrid zones are most likely recent Holocene contacts following the rapid demographic and spatial expansion of populations from these southern Mediterranean refugia [30,31,32]. Genetic and morphological data have enabled the accurate definition of the contact zones for certain groups in Central and Northern Europe in the Western Palearctic [22]. However, knowledge of the location and dynamics of contact zones within the five main refugia of the Mediterranean Basin remains limited.

Türkiye (Anatolia and Thrace) is one of the world’s rare regions in that it hosts three different biodiversity hotspot regions (the Mediterranean, Iran–Turan, and Caucasus) [33] and is also an important ancestral and genetically diverse area for temperate-adapted (Mediterranean), cold-adapted (Alpine–Europe–Siberian), and humid-subtropical (Colchian) forms in the Palearctic [34,35,36,37,38,39,40,41]. The heterogeneous topography and changing climatic features across short distances have led to the formation of many allopatric refugia in Türkiye. In addition, its unique geographical structure, surrounded by high mountain ranges and water bodies, gives it an intra-continental island characteristic. Based on these topographic and climatic features, an increasing number of studies have concluded that Türkiye has high potential for cryptic microrefugia and mosaic hybrid areas [36,42,43,44]. 

Türkiye’s geographic features (rivers, straits, distance from the sea, etc.), heterogeneous topography (mountain chains and elevation), and the areas of intersection of different climatic zones serve as possible soft and hard barriers restricting the dispersal capability of populations [45]. Depending on their permeability, altitudinal barriers (mountains) have played an important role in shaping Anatolian biodiversity during the Quaternary climatic fluctuations [46,47]. Evidence for this is the presence of many closely related species in Türkiye near high altitudinal (hard) barriers [42,48,49,50,51,52,53]. The role of an altitudinal barrier as a contact zone or phylogenetic breaking area is closely related to its hard or soft barrier effect during climatic fluctuations [54,55]. Since hard barriers prevent gene flow between closely related populations, they are generally associated with phylogenetic breaking [56,57]. Unlike hard barriers, the effects of permeable/soft barriers can temporarily change due to diverse factors such as climate, geography, and tectonics. Therefore, parapatric structures, hybridization, and introgression events are most likely to be observed around permeable barriers [8,58,59]. This renders them and their vicinities the most suitable regions for detecting hybrid zones within the distribution areas of closely related lineages [55]. In contrast, if altitudinal barriers have continuously maintained a hard barrier effect, their regions will be less likely to host hybrid zones [60]. Türkiye has many moderately high mountain chains (1.500–2.500 m), which means that they may have had a soft or hard barrier effect during past climatic fluctuations in Anatolia. Therefore, hybrid zones in Türkiye are likely to be observed around these permeable barriers.

Türkiye’s topography rises gradually from west to east, creating a clinal climate zone throughout the landmass [45,61]. During the Quaternary period, these altitudinal differences induced distinct glaciation dynamics in eastern and western Türkiye [62]. In particular, Western Türkiye did not experience dramatic glaciation events during the LGM (~21 kyBP), except for a few highlands [13]. In contrast, eastern Türkiye experienced intense glaciation and permafrost during the LGM [63,64]. These different climatic dynamics shaped the distinct demography and distribution patterns of populations in each region [45,65]. The Anatolian Diagonal is one of the important phenomena for Türkiye’s biogeography, and it is a kind of biogeographic border between the eastern and western parts of Türkiye [66,67]. Its formation follows a northeast–southwest direction, being superimposed on Anatolia’s gradual west–east uplift chain [66,67]. Depending on ongoing climatic conditions, these elevation differences along the Anatolian Diagonal caused a barrier effect on many lineages [68,69]. Therefore, the Anatolian Diagonal and its surrounding area might be an active secondary contact zone for lineages isolated in eastern and western Türkiye during glacial periods.

Due to its high ecological tolerance and wide distribution range, *A. chukar* is a good model organism for studying potential cryptic contact zones in Türkiye. Both natural and artificial/human-mediated hybridizations are common in the genus *Alectoris*. Several natural hybridizations have been reported: *A. chukar* × *A. magna* on Liupan Mountain in China (Gansu Province) [70,71,72,73]; *A. rufa* × *A. greaca* in the French Alps [23,25,74,75]; and *A. greaca* × *A. chukar* in the Thrace–Balkans region [76]. Given that the first two are also well-documented by genetic data, Türkiye’s Thracian region can be assumed to be a possible natural contact area for *A. greaca* × *A. chukar*. The long-standing historical trade of *A. chukar* individuals along the Silk Road has made it one of the most frequently introduced species of the genus. In Europe, the native species *A. rufa* and *A. greaca* have been intensely contaminated by China clade-B *A. chukar* genes, which may have caused the introduction of non-native genes and a loss of local adaptation, and contamination events originating from *A. chukar* in Europe occurred at least twice, derived from different sources [24,30,31,77,78,79,80]. However, no genetic data have indicated any contamination events in Europe originating from Türkiye’s *A. chukar* populations. Althoughthe presence of China clade-B contaminations in Türkiye was reported in [79], this study only accessed three samples from the southeastern part of the country; hence, it did not cover most of the species’ distribution area in Türkiye. Thus, given the lack of adequate genetic studies on *A. chukar* populations, the level of contamination of *A. chukar* populations in Türkiye due to the human-mediated introduction of China clade-B individuals and congenerics cannot yet be estimated. 

Accordingly, in the present study, 2 mitochondrial (D-loop and Cyt-b) and 13 polymorphic microsatellites markers were analyzed using 368 *A. chukar* samples drawn from 16 different parts of Türkiye. The study aimed to determine (i) the geographic genetic structure of *A. chukar* populations in Türkiye, (ii) a possible hybrid area of *A. chukar* populations near the Anatolian Diagonal, (iii) whether there is a natural *A. greaca* × *A. chukar* hybrid area in Türkiye’s Thracian region, (iv) whether the Turkish strait system (the Çanakkale–Marmara–İstanbul water body) played a barrier role that limited the dispersal of *A. chukar* populations, and (v) the presence of congeneric-originating, human-mediated gene flow in Türkiye.

## 2. Materials and Methods

### 2.1. Sampling and DNA Extraction

**Figure 1:**
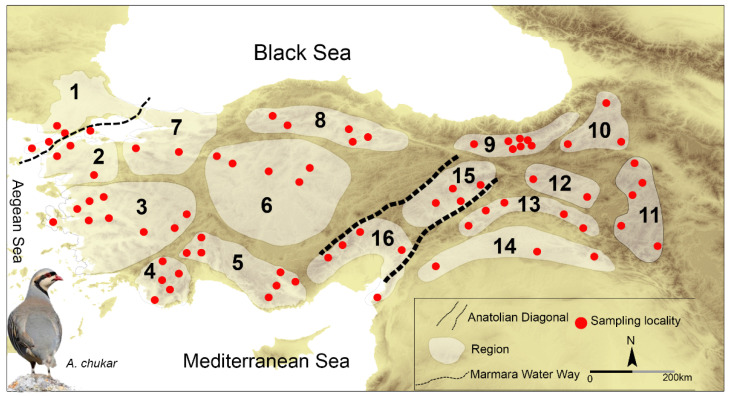
Sampling areas of *A. chukar* in Türkiye.

Given the species’ ecological characteristics and topographical heterogeneity of Türkiye (between Thrace and Anatolia), field surveys were conducted in 16 different sampling areas/hypothetical populations between 2017 and 2018. A total of 368 fresh tissue and blood samples were collected from 73 different locations in the 16 areas representing the major distribution area of the *A. chukar* in Türkiye (Figure 1, SMS1: Table S1). Each sampling area was also considered as a different population. The identification and descriptions of specimens were performed based on the taxonomic and morphological characters of *A. chukar* [81,82]. After morphological examination, the fresh tissues and blood samples were placed in 96% ethanol in the field and then stored at −20 °C in the laboratory of Burdur Mehmet Akif Ersoy University.

Total DNA extractions were performed from the blood and muscle tissues using a GeneJET Genomic DNA Purification Kit (Thermo Scientific #K0722, Waltham, MA, USA), following the manufacturer’s protocol. The DNA samples were then stored in 96% ethanol at −20 °C. Microsatellite markers and mtDNA regions (D-loop and Cyt-b) were amplified by polymerase chain reaction (PCR). The primer pairs and PCR conditions are described in SMS2: Table S2. 

### 2.2. Analysis of mtDNA

#### 2.2.1. Sequence Alignment and Data Preparation

For pairwise alignment, the AB1 files of the D-loop and Cyt-b regions were manually edited and visually controlled using SEQUENCER v4.1.4 (Gene Codes Corporation, Ann Arbor, MI, USA). Since the nuclear genome has multiple copies of mtDNA (Numt—nuclear mitochondrial pseudogene) [83,84], the DNA sequences were screened for the probability of being Numt copies. Scanning for the Cyt-b gene was performed using DNASP v.6 [85] by checking for the presence of a stop codon, insertion, and deletion in the sequence. After the pairwise alignments and rough data verification by eye, the sequences of each mitochondrial region were converted into separate fasta files for use in the analyses. The sequences of unique haplotypes for D-loop and Cyt-b were deposited in GenBank (see the accession numbers in SMS2: Table S3).

Phylogenetic analyses were conducted by downloading 262 sequences for D-loop and 365 sequences for Cyt-b of the genus *Alectoris* from Genbank (NCBI: National Center for Biotechnology Information) and combining the haplotypes from these sequences with our datasets. To identify any genetic contamination of *A. chukar* populations with congenerics and China clade-B, phylogenetic analyses were conducted using combined data matrices. For the phylogenetic analyses, *Coturnix coturnix* (Genbank accession No.: L08377) and *Tetraogallus himalayensis* (Genbank accession No.: NC027279) were used as outgroups for Cyt-b, while *Coturnix japonica* (Genbank accession No.: NC003408) and *Tetraogallus himalayensis* (Genbank accession No.: NC027279) were used for D-loop. To identify *A. chukar*’s geographic genetic structure in Türkiye, only the data matrices that included *A. chukar* sequences obtained in this study were used. For the data analysis of these sequences, *A. philbiyi* (Genbank accession No.: Z48774 and AJ222737 for Cyt-b and D-loop, respectively) and *A. magna* (Genbank accession No.: EU839473 and AJ222732 for Cyt-b and D-loop, respectively) were used as outgroups. Due to the absence of some *Alectoris* sequences from different localities in the NCBI, all phylogenetic analyses were performed separately for the D-loop and Cyt-b regions. The data matrices were prepared using MEGA v.7 [86], while the multiple sequence alignments were carried out using the online version of MAFFT v.7.245 [87] “https://mafft.cbrc.jp/alignment/server/ (accessed on 8 August 2022)”, following the default strategy. Redundant sequences and haplotype frequencies were calculated using DNASP v.6 for the Cyt-b and D-loop regions, respectively.

#### 2.2.2. Phylogenetic Analyses

The substitution models for the D-loop and Cyt-b data matrices were calculated using JMODELTEST v.0.1 [88]. The most suitable substitution models were selected based on Akaike and Bayesian information criteria. All matrices were analyzed with the Bayesian inference (BI), maximum likelihood (ML), and maximum parsimony (MP) approaches. The analysis of Genbank and our dataset was conducted with 142 haplotypes (of two outgroups) for D-loop and 90 haplotypes (of two outgroups) for Cyt-b, together with the outgroups. The most suitable models were HKY+G (0.1710) for D-loop and TPM2uf+G (0.1940) for Cyt-b. With the selected substitution models, Bayesian inference (BI) analyses were run in parallel with two different simulations (nrun = 2), ngen = 9.000.000 for Cty-b and ngen = 5.000.000 for D-loop, samplefreq = 100 printfreq = 1.000 nchains = 4, savebrlens = yes, performed using parameters. 

The phylogenetic analysis was performed with our dataset using 171 haplotypes for D-loop and 63 haplotypes for Cyt-b. The most suitable models were GTR+I (0.8370)+G (0.5400) for D-loop and K80+G (0.0110) for Cyt-b. Using these substitution models, BI analyses were run in parallel with two different simulations (nrun = 2), ngen = 9.000.000 for Cty-b and ngen = 5.000.000 for D-loop, samplefreq = 100 printfreq = 1000 nchains = 4, savebrlens = yes, performed using parameters. The BI analyses were run using MrBayes v3.1.2 [89] with the parameters. During the analysis, the software tool TRACER v.1.5 [90] was used to monitor the effective sample size, the parameters, and the number of generations. The posterior probabilities for each branch of the majority-rule consensus trees were calculated with the first 20% of trees as burn-in. The ML analyses were conducted with RAxML [91] using the ML+rapid bootstrap options, with 106-bootstrap resampling using the GTRGAMMAI model. The maximum parsimony (MP) analysis was conducted using PAUP v. 4.10b [92], with 10 random additions following the heuristic search approach and the tree bisection–reconnection (TBR) algorithm. To assess the branch confidence, 100-nonparametric-bootstrap resampling was run using the same program [93]. Figtree v.1.4.4 [94] was used to visualize the results.

### 2.3. Geographic Genetic Structure

**Figure 2:**
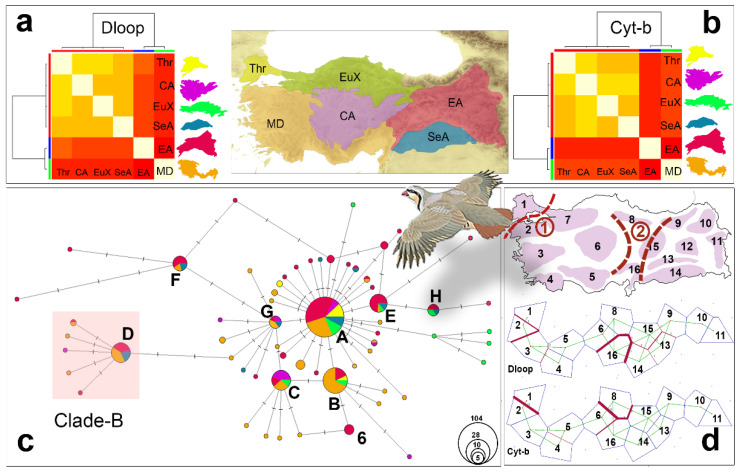
Geographic genetic structure of haplotypes. Results of heatmap analyses: (**a**) Dloop and (**b**) Cty-b. The map of six geographic regions in Türkiye: Thr: Thrace, MD: Mediterranean, EuX: euxinic, Ca: Central Anatolia, EA: East Anatolia, SeA: Southeast Anatolia. (**c**) Cty-b haplotype network. (**d**) Barrier analyses. The dashed lines marked as 1 and 2 represent the Marmara waterway (1) and the Anatolian Diagonal (2).

To determine the effect of Türkiye’s geography on the genetic structure of *A. chukar* populations, phylogenetic and population genetic analyses were conducted based on the six different geographic regions (Figure 2). These regions were defined in terms of the country’s geographic structure, climatic zones, and altitudinal heterogeneity. To better understand the genetic diversity distribution of *A. chukar* populations in Türkiye, haplotype network analyses were carried out with 61 haplotypes for Cyt-b and 115 haplotypes for D-loop using NETWORK v.4.5.1.6 [95]. The median-joining algorithm with default settings was used to construct the network (weight = 10 and e = 0). Regional unique haplotype diversity can help to identify possible refugial and diversification areas for *A. chukar* populations in Türkiye. To do this, heatmap cluster analyses were performed based on the number and frequency of unique and shared Cyt-b and D-loop haplotypes in each region. The analyses were run in R using the *apcluster* package [96].

The number and frequency of shared haplotypes among the six geographic regions can reveal genetic relationships between *A. chukar* populations and possible geographic barriers. Therefore, principal coordinate analyses (PCoA) were performed with the shared haplotypes. Pairwise F_ST_ estimations were conducted using ARLEQUIN v.3.5.2.2 [97] with Kimura’s two-parameter model (1980), defining 100 permutations for significance, 1.000 permutations for the Mantel test, and a significance level of 0.05. PCoA analyses were conducted using GenAlEx v.6.5b3 with the covariance-standardized method [98].

The locations of possible genetic barriers that have restricted gene flow between *A. chukar* populations were explored in BARRIER v.2.2 [99] using genetic distance matrices (F_ST_). The program uses a geometric approach to compute barriers on a Delaunay triangulation. In the analysis, one calculates the location and direction of barriers and provides a visual representation of how the landscape impacts population dispersal. The areas with the largest genetic differences between population pairs were identified using Monmonier’s algorithm. The significance of these barriers was then assessed by a bootstrap test using 100 F_ST_ matrices, simulated in GenPOP embedded in DIYABC v.2.1.0 [100] with a suitable substitution model, carried out separately for the D-loop and Cyt-b data. Following the simulations, F_ST_ values for each gene region were calculated using the simulated data matrices in ARLEQUIN. Barrier analyses were performed with 14 populations using the D-loop and Cyt-b regions, respectively. Due to the inadequate sample size of populations 7 and 12, the Barrier analyses were performed with 14 regions.

### 2.4. Population Genetics and Demographic Analysis

The genetic diversity and historical demographic parameters of the 16 populations were estimated separately using the D-loop and Cyt-b sequences. ARLEQUIN was used to estimate the number of unique haplotypes without gaps (k) and with gaps (K), haplotype diversity (h), the number of segregating sites (s), nucleotide diversity (π), mean number of pairwise differences between the k haplotypes (d) of each population, and pairwise F_ST_ values. Heatmap plots were created using the *apcluster* package in R, using the pairwise F_ST_ values for D-loop and Cyt-b separately to evaluate the significance of the genetic differentiation between the 16 populations.

To determine whether past climatic fluctuations affected the demography and population structure of *A. chukar,* neutrality tests and mismatch distribution analyses were performed using the D-loop and Cyt-b regions separately. Historical population expansions and bottleneck events were estimated using Tajima’s D [101] and Fu’s Fs [102] tests. Historical demographic patterns were assessed by mismatch analysis using a model of stepwise population expansion. The demographic expansion parameters τ, θ_0_, and θ_1_ were estimated using a generalized nonlinear least-squares approach. The overall validity of the observed and expected mismatch distributions were evaluated using the tests of Harpending’s raggedness index (Hri) [103] and the sum of squared differences (SSD) [104]. The significance of Hri and SSD were assessed by parametric bootstraps (10.000 replicates), with the significant values taken as evidence of a departure from the estimated demographic model of sudden population expansion. All these parameters were estimated using ARLEQUIN.

To test the robustness of the results obtained from the demographic analyses, a coalescent-based analysis was performed for all populations. The population growth rate parameter g was estimated using LAMARC v.2.1.3 [105], which uses a Metropolis–Hastings MCMC algorithm to identify the demographic expansion parameter values. For each phylogroup, the following strategy was applied: 10 short chains of 1.000 steps and 2 long chains of 10.000 steps, sampling every 20th step, and a burn-in of 1.000 trees. To estimate and visualize the past demographic structure of the *A. chukar* populations, GMRF Bayesian skyline plot (BSP) analyses were performed [106,107]. Employing an MCMC and coalescent-based method, the GMRF Bayesian skyline plots (BSPs) were used to estimate the changes in the effective population size from the starting time to the most recent common ancestor (TMRCA). BSP analyses were carried out for each *A. chukar* population (except for 7 and 12) in BEAST based on the Cyt-b sequences. A strict molecular clock model and Bayesian skyline tree prior were applied as sequence substitutions, which were expected to be constant for each intraspecific lineage. Chains for 100 million generations or more were run until the effective sample size (ESS) was > 200, discarding the first 10% as burn-in. The results were summarized and displayed in TRACHER 1.6.

The asymmetric migration rates (M) and gene flow direction between the six regions were estimated using the Cyt-b matrix through Bayesian Markov Chain Monte Carlo (MCMC) coalescent modelling implemented with the LAMARC-migrate module. Uniform priors were placed on Θ [0.001–1] and M [1–800]. Asymmetric migration rate analyses were run with an automatically adjusted heating temperature using four initial chains and two multiple replicates of 10.000 iterations with a burn-in of 1.000. The convergence likelihood for the MCMC chains was verified using TRACER.

### 2.5. Microsatellite Genotyping and Analysis

The primers of 13 polymorphic microsatellite markers and their amplification conditions are summarized in SMS2: Table S2. Fragment analyses were performed using the 500 LIZ size standard with an ABI 3730xl 96-capillary DNA analyzer (Macrogen Inc., Amsterdam, The Netherlands). The results were transferred as .fsa files, while the fragment size was scored using the GENEIOUS prime program by selecting the 500 LIZ size standard. After scoring the marker sizes, the matrices were converted into appropriate analysis files using CONVERTER v.1.31 [108]. The missing value ratio for each locus and population was calculated using the *adegenet* [109] package in R. Descriptive statistics and the exact Hardy–Weinberg test (HWE), used to assess deviations from the Hardy–Weinberg equilibrium (HWE) for each locus, was performed with ARLEQUIN. The pairwise genetic distance FST values between the populations were calculated using the *poppr* [110] package in R.

To determine the genetic structure of *A. chukar* populations in Türkiye, two different clustering-based analyses were performed. First, a discriminant analysis of principal components (DAPC) was performed using *adegenet*, which uses sequential K-means and model selection to infer genetic clusters so as to identify and describe the clusters of genetically related individuals. Second, Bayesian MCMC clustering analysis was carried out using STRUCTURE v. 2.3.4 [111] to detect the genetic structure patterns of *A. chukar* in Türkiye. STRUCTURE was run assuming correlated allele frequencies and admixture, without using prior population information [112]. Ten independent runs were performed for each value of K = 1–10 to estimate the ‘true’ number of clusters with 500.000 MCMC cycles, following a burn-in step of 100.000 iterations. The admixture model with the correlated allele frequencies was applied with 500.000 Markov chains and lengths of 100.000. The numbers of populations (K) were set as 2–10, and each K value was run 10 times. The most appropriate K was identified according to the ΔK method [113] and defined using log probabilities [Pr(X|K)]. The STRUCTURE HARVESTER [114] and CLUMPAK “http://clumpak.tau.ac.il (accessed on 17 August 2022)” [115] online servers were used to calculate the most appropriate K value. CLUMPP [116] was used to summarize the replicated results, while DISTRUCT [117] was used to produce the bar plot for the combined results. To understand the microsatellite-based genetic relationship between the *A. chukar* populations, Nei distance dendrograms [118] were constructed using the POPGENE32 v.2.8.3 package. Due to their small sample sizes, populations 7 and 12 were excluded from the dataset for the Nei analysis.

### 2.6. Estimating Divergence Time and Gene Flow: ABC

To test the competing alternative hypotheses regarding the divergence time and admixing events among *A. chukar* populations in Türkiye, approximate Bayesian computation (ABC) analyses were performed using the microsatellite dataset of the 16 populations [119]. This type of analysis identifies the most probable of the alternative hypotheses by comparing the observed (priors) and simulated parameters with each other. The most probable hypothesis was determined using three different evaluation processes: (i) direct estimation (by calculating the final probability values of each hypothesis), (ii) logistic regression, and (iii) PCA analysis, calculated by considering 1.000 simulations distributed in the observed data [120,121].

**Figure 3:**
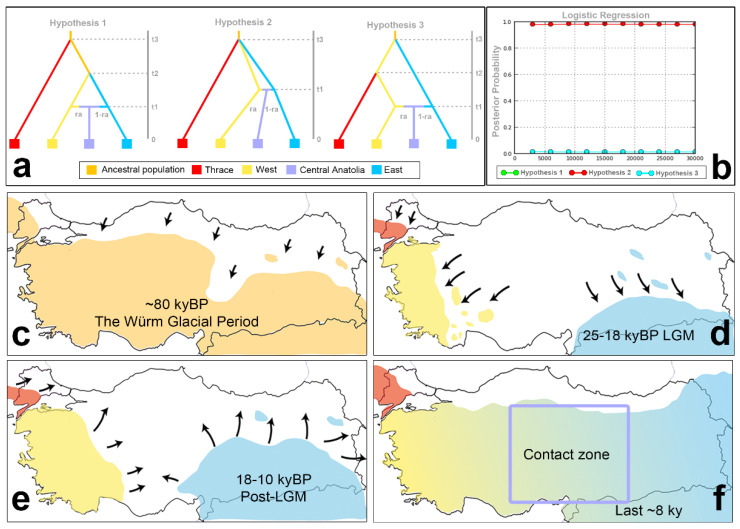
The ABC analysis results. (**a**) The comparison of the three hypotheses using the DIYABC approach for the four clusters of *A. chukar* identified in the cluster analyses in Figure 4 and Figure 5. These clusters are Thrace: population 1, West: populations: 2, 3, 4, and 5, East: populations: 9, 10, 11, 12, 13, and 14, and Central Anatolia: populations: 6, 7, 8, 15, and 16. In these hypotheses, t: time in generations, N: effective population sizes of the different populations denoted by different colors, and r: immigration rates originating from the donor population. (**b**) The logistic regression plot for each simulated hypothesis, plotting the posterior probabilities (*y*-axis) by the number of simulations used to calculate it (*x*-axis). (**c**–**f**) The scenarios supported by the ABC analysis.

Considering the analysis results of the mtDNA and microsatellite markers, three hypothetical evolutionary hypotheses were built regarding the divergence/mixing time and gene flow direction/rate of *A. chukar* populations (Figure 3). For the ABC analysis, China-clade-B-contaminated individuals were excluded from the dataset to avoid misestimation of the parameters. All analyses were conducted with 13 microsatellite loci from 329 individuals based on constant effective population sizes. To explain the formation of *A. chukar*’s present geographic genetic structure in Türkiye, we suggested three hypotheses based on the Pleistocene climatic conditions, topographic heterogeneity, and geographic structure. These hypotheses were based on four geographic genetic areas: Thrace (population: 1) and East (populations: 9, 10, 11, 12, 13, and 14), West (populations: 2, 3, 4, and 5), and Central Türkiye (populations: 6, 7, 8, 15, and 16).

The first hypothesis predicts that the Thrace population diverged from the other ancestral *A. chukar* populations at time t3 before the East and West populations diverged from each other at time t2. The second hypothesis predicts the simultaneous divergence of all populations at time t3. The third hypothesis predicts that the East and West populations first diverged at time t3, when the West and Thrace populations were still in contact. At time t2, the Thrace population was split from the West population. All three hypotheses also predict that an admixture event occurred between the East and West populations at time t1, which produced a new mixed population in Central Anatolia. The admixture rate parameter (ra) represents the gene flow from the West population to the Central Anatolian population (Figure 3 presents the three hypotheses).

The priors required for the ABC analysis were estimated from the observed data (Table 1, SMS6: Table S14). The analysis was conducted using DIYABC v.2.1.0 [100] based on a total of 329 individuals: 140 from the East, 72 from the West, 100 from Central Anatolia, and 17 from Thrace. The three hypotheses were written using the DIYABC program’s coding language. The analysis was run using 10^6^ simulated datasets per hypothesis, considering a 1:1 female-to-male sex ratio, a generalized stepwise mutation model (SMM), and uniform priors with default values for all the parameters. The analyses were performed with 13 parameters and 42 summary statistics. For the time estimation, the number of generations was taken to be 3.9 years/generation [122].

To assess the posterior probability of each hypothesis, a weighted polychotomous (multinominal) logistic regression analysis was performed. The analysis was conducted using 1% of the simulated datasets closest to the observed data [100,123,124]. The 500 simulated pseudo-observed datasets (PODs), in each scenario, were used to estimate type I and type II error rates and assess the degree of confidence in the hypothesis choice [125]. The posterior distribution value for all the parameters of the best-supported hypothesis was estimated using local linear regressions on 1% of the simulations closest to the observed data after a logit transformation of the parameter values [100,123,126]. The performance of the parameter estimation was determined by calculating the relative median of the absolute errors (RMAE) from 500 PODs [127]. The goodness of fit of the best-supported hypothesis was evaluated using 104 simulated PODs from the posterior distribution of all the parameters. PCA was applied to visualize the fit between the simulated and observed datasets.

### 2.7. Ecological Niche Modelling and Past Distribution Change 

Ecological niche modelling (ENM) analysis was performed to determine whether the current geographic distribution pattern of *A. chukar* populations in Türkiye was shaped in response to Pleistocene climatic fluctuations. The ENM analyses were performed in MAXENT v.3.3.3k [128,129] based on the maximum entropy approach. To estimate the current and LGM distributions of *A. chukar*, the analyses were run with 19 bio-climate variables from WorldClim during two periods: the Present (1960–1990 AD) and the LGM (0.021–0.018 Ma) “http://www.worldclim.org (accessed on 2 September 2022)” [130,131]. For the LGM (c. 21.000 yearsBP), the climate data were derived from the Community Climate System Model (CCSM) [132] and the Model for Interdisciplinary Research on Climate (MIROC) [133]. The General Circulation Model (GCM) data were downloaded with a spatial resolution of 30 arc-sec for the Present and 2.5 arc min for the LGM. All pre- and post-MaxEnt data analyses were performed in ArcGIS v10.7.1) and SDMToolbox v.2.0 [134]. Before this, a correlation analysis of all 19 bioclimatic predictors was performed to identify any highly correlated (r > 0.8, r < −0.8) bioclimatic layers. To exclude highly correlated and redundant climatic variables from the climatic datasets, Pearson correlation tests were performed on the bioclimatic layers using SDMToolbox. Since elevations in Türkiye above 1.600 m (and above 1.200 m in the same locations on the northern slopes) had a permanent snow layer during the LGM, 21 geographic reference points higher than 1.600 m were excluded from the ENM analysis. The ENMs were conducted based on 53 geo-reference points for the LGM and 73 for the Present (SMS1 Table S1). The data analyses were run in the Maxent program using 25 random test percentages, 50 replicates, and 5.000 iterations, selecting the minimum training presence threshold rule and subsample run type.

## 3. Results

### 3.1. Phylogenetic Relationships

From the 368 samples, 273 sequences for D-loop and 277 sequences for the Cyt-b regions were obtained (SMS2: Tables S4–S6, SMS4: Tables S7–S9). For the Cyt-b regions, 90 haplotypes were identified from 312 sequences (277 from the study + 33 haplotypes (out of 365 NCBI sequences, Genbank + 2 out groups)) with a length of 1014 bp. Of the 1014 sites, 729 were constant and 285 were variable (121 singleton and 50 parsimony-informative), and no indels were detected in the sequences. No shared haplotype was detected between *A. chukar* and other congenerics in Türkiye. Phylogenetic analyses of the 90 haplotypes supported the ML, MP, and BI trees, with similar topologies and branch support (SMS3: Figure S1). The Cyt-b gene trees provided significant statistical support to the monophyly of the genus, with seven different species in the genus (*A. melanocephala*, *A. barbara*, *A. rufa*, *A. graeca*, *A. philbyi*, *A. magna*, and *A. chukar*). All analyses identified *A. melanocephala* and *A. barabara* as the most ancestral lineages, whereas *A. rufa*, *A. graeca*, *A. philbyi* + *A. magna*, and *A. chukar* were identified as different species. The Cyt-b tree supported the presence of A and B clades within *A. chukar*. Although the monophyly of clade-A was supported by the low bootstrap/posterior probability, China clade-B appeared to be paraphyletic. Only three subspecies’ sequences from China clade-B (*A. c. potanini*, *A. c. pubescens*, and *A. c. falki*) exhibited a monophyletic pattern. Six haplotypes representing 22 individuals were in China clade-B (AntC1, VanC, AntC2, AntC3, AntC4, and AntC3) but collected from Türkiye. Although this result supports the presence of China clade–B contamination in Türkiye, no congeneric or other species-related contaminations were detected. Similar results were obtained from the Cyt-b trees constructed using only the *A. chukar* haplotypes (SMS3: Figure S2; for D-loop phylogeny, see SMS3: Figures S3 and S4).

### 3.2. Geographic Genetic Structure of Haplotypes

The bifurcated phylogenetic trees of the Cyt-b and D-loop regions did not resolve the phylogenetic relationships between the haplotypes within Türkiye. Therefore, network analyses were performed separately for both the Cyt-b and D-loop regions. The phylogenetic relationships between the haplotypes were evaluated in terms of the six main geographic regions in Türkiye. The results of the Cyt-b region network analysis with 61 haplotypes revealed a star-like phylogeny for the Anatolian *A. chukar* populations (Figure 2c). The majority of Cyt-b haplotypes were concentrated around the eight main haplotypes that were separated from the ancestral haplotype by only one or two mutations. The haplotype pattern in the network analysis indicated a sudden demographic expansion of the Anatolian *A. chukar* population (Figure 2c). The haplotypes from the Mediterranean and Eastern Anatolia were dominant in the analysis, while unique haplotypes from Thrace, Central Anatolia, and the euxinic regions were also detected. Although China clade-B haplotypes were found in the Mediterranean and Central and Eastern Anatolia, no contamination was observed in Thrace or the euxinic regions. The haplotype sharing pattern indicated intense mixing events within different parts of Anatolia. Haplotype A, an ancestral haplotype, was the most widely shared haplotype among the six main geographic regions, followed by the B, C, and G haplotypes. The haplotypes derived from the B, C, and G haplotypes were mainly found in the Mediterranean region, whereas the E, H, and F haplotypes and their derivatives were mostly observed in Eastern Anatolia. Haplotype F and its derivatives formed the haplogroup farthest from the center of the haplotype network, being even more distant than the clade-B haplotypes. This result supports the claim that Anatolia was the ancestral area of the species in the Palearctic. Haplotype D and its derived haplotypes together represented the China clade-B contaminations (Figure 2c), which were detected in 23 of the 277 Cyt-b sequences, representing 8% of the total sequences. Of these, 11 were from the West and 12 were from East Anatolia. The heatmap-cluster analysis performed with 45 unique Cyt-b haplotypes from the six geographic regions confirmed that the Mediterranean (West) and Eastern Anatolia were major diversification areas for *A. chukar* populations in Türkiye (Figure 2b). The heatmap cluster analysis for the D-loop region also supported the claim that the Mediterranean and Eastern Anatolia were the main diversification areas for *A. chukar* populations in Türkiye (Figure 2a). For the D-loop region network analysis, see SMS3: Figure S5 and SMS2: Table S6. 

The Cyt-b PCoA analysis, conducted using the pairwise F_ST_ values of the six geographic regions, excluding the clade-B haplotypes, indicated a remarkable geographic genetic structure of the Anatolian *A. chukar* populations (SMS3: Figure S5 and SMS2: Table S6). The PCoA analysis showed that there are three geographic groups in Türkiye: Thrace; the Mediterranean and euxinic; and East, Southeast, and Central Anatolia. Thrace was genetically the most distant within *A. chukar*’s distribution area, whereas the Mediterranean region was genetically the closest geographic area to Thrace. 

The barrier analyses of both D-loop and Cyt-b suggested that there are roughly two geographic barriers in *A. chukar*’s distribution area in Türkiye (Figure 2d). One is the Marmara waterway, comprising the Turkish Straits and the Marmara Sea, which creates a strong water barrier for many terrestrial organisms. In Figure 2, the barrier effect of this waterway is indicated by the thick lines drawn between populations 1 and 2 based on both analyses. Although the second barrier’s location was not clearly indicated in either analysis, the lines in Figure 2d roughly represent the location of the Anatolian Diagonal. 

### 3.3. Genetic Diversity, Historical Demography, and Migration Rates

To determine the current genetic diversity of *A. chukar* populations in Türkiye and the effect of the Quaternary climatic fluctuations on the species’ past historical demography, descriptive statistics and demographic analyses of the 16 populations were conducted with the mitochondrial Cyt-b and D-loop regions. The genetic diversity parameters calculated with the Cyt-b data indicate high genetic diversity parameters in all the populations, except for the Thrace population/population 1 (SMS4: Table S7). The descriptive statistics indicate that populations 2 and 11 have the highest haplotype diversity, while populations 4 and 10 have the highest nucleotide diversity and mean number of pairwise differences between haplotypes. Populations 1 and 16 have the lowest values for all the diversity parameters. Population 1 (Thrace) has the lowest value of pairwise haplotype distance (SMS4: Table S7), which suggests that the haplotypes in population 1 are separated by only a few nucleotide differences. The small population size and lack of gene flow with other populations indicate that the Thracian population has faced high inbreeding rates. In the Cyt-b dataset, clade-B contaminations were detected in 1 individual in populations 6 and 10, 2 individuals in populations 3 and 11, 3 individuals in population 14, 5 individuals in population 13, and 8 individuals in population 4. The highest clade-B contamination was found in population 4. After excluding China clade-B haplotypes from the populations, population 2 had the highest genetic diversity parameters, followed by populations 10, 11, and 14. These four regions act as gateways to other eco-geographical areas, namely, the Caucasus, Iran, the Arabian Peninsula, and the Levant. The high values of the mean pairwise differences obtained for populations 10, 11, and 14 indicate that these three regions may be mixing areas or refugia for *A. chukar* populations in the East.

To investigate the past demographic structures of Türkiye’s *A. chukar* populations, neutrality tests and g parameter and mismatch distribution analyses were performed with the Cyt-b dataset (Table 1, SMS4: Figures S6 and S7), using GMRF Skyride analysis with the Cyt-b region to visualize the populations’ time-dependent demographic patterns (SMS4: Figure S7). The neutrality test results for the Cyt-b region revealed that the Tajima’s D parameters supported a weak, sudden population expansion model only for populations 9 and 16 (Table 1). On the other hand, the Fu’s Fs analyses estimated a remarkable demographic expansion of all the populations except for 1, 6, and 10. The most prominent expansions were observed in populations 9, 14, and 15. The g parameter, which is used to calculate the population expansion through the Bayesian MCMC algorithm, supported an expansion of all the Anatolian populations. In contrast to the neutrality tests (Tajima’s D and Fu’s Fs), the Bayesian analyses indicated a strong demographic expansion of populations 1 and 10 and a weak expansion of population 6. The observed and expected distribution plots of the mismatch distribution analysis supported the sudden expansion model of *A. chukar* populations and indicated mixing events in populations 2, 4, 5, 6, 10, and 13. (SMS4: Table S10, SMS4: Figure S7). 

The past demographic expansion time estimations were conducted using tau (τ) values for those populations that showed a strong sudden expansion model. The estimations were performed using Cyt-b haplotypes by removing the clade-B haplotypes. The time estimations for populations 2–5, 9, 13–15, and 16 indicate that the demographic expansion of these populations began in Anatolia at approximately 8–22.7 kyBP (Table 1). Considering the distribution of *A. chukar* in Anatolia, the demographic expansion times correlate with the elevation and north–south location of populations in Anatolia. Because the Eastern Anatolian populations are located at higher altitudes than the Western populations, the demographic expansion time of the Eastern populations (average 15 kyBP) is more recent than that of the Western populations (average 17 kyBP) (Table 1). The demographic expansion times were strongly negatively correlated with the altitude of the regions (CorSpearman’s rho = −1.00, *p* < 0.01, N:4). The demographic expansion time range indicates that the population growth of the *A. chukar* population in Western Anatolia (14–23 kyBP) began at the end of the LGM (~22 kyBP), while the population growth in Eastern Anatolia (12.5–16 kyBP) began during the post-LGM period, particularly at the end of the short ice age or Younger Dryas (~12.8–~11.6 kyBP) (Table 1).

### 3.4. Microsatellite Genotyping

In total, 347 individuals from 16 populations were successfully genotyped with 13 microsatellite loci. The overall number of alleles per locus ranged from 3 to 17 (SMS5: Table S1). A total of 164 alleles were detected, with a mean value of 5.75 alleles (ranging from 1.63 to 16.81, SMS5: Table S11). Populations 16, 15, and 13 had the highest mean number of alleles per locus, while populations 12, 7, 2, and 1 had the lowest. Among the loci, the locus with the highest missing data value (34%) was MCW0069, while the highest missing value among all the populations was that of population 9 (41%) (SMS5: Figure S8). The total missing value of the data was 22%. The observed heterozygosity (HO) was lower than the expected heterozygosity (HE) in all the populations. The highest expected heterozygosity (He) values were detected in populations 9 (0.685), 10 (0.661), 16 (0.651), and 13 (0.657), while the lowest value was detected in population 1 (0.498) (SMS5: Table S12). The F_IS_ values were significant for all 14 populations, with the highest value in population 1 (0.3439) and the lowest in population 16 (0.0023). Among the 14 populations, population 1 had the lowest values for almost all the parameters, which suggests that population 1 may have undergone a recent bottleneck. The pairwise F_ST_ result indicated that population 1 is the most distinct population. In addition to population 1, populations 3, 4, and 5, which represent the Western populations, showed prominent differences from the other populations (SMS5: Figure S9).

### 3.5. Population Structure

**Figure 4:**
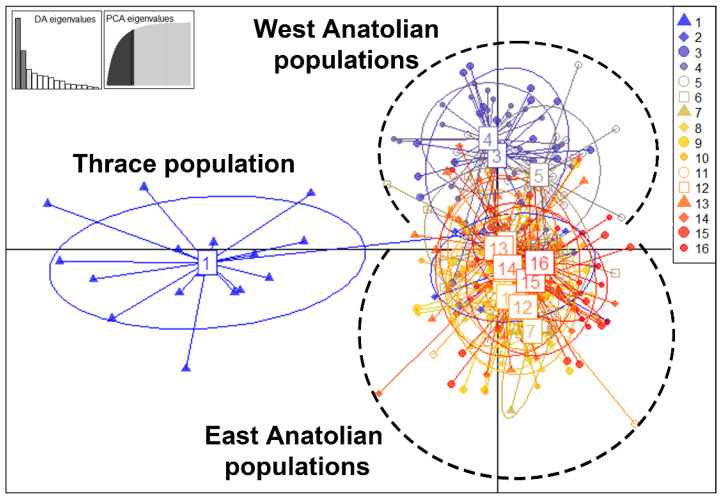
Discriminant analysis of principal components (DAPC) results based on 13 microsatellite loci studied among 347 individuals from 16 *A. chukar* populations.

The results of the multidimensional genetic distance test, a discriminant analysis of principal components (DAPC) performed on 347 *A. chukar* individuals, supported the existence of three genetic clusters in Türkiye (Figure 4). Of these, clusters 2 and 3 partially overlap, while cluster 1 is clearly distinct from them. Cluster 1 includes only the Thracian population, while cluster 2 includes populations 2, 3, 4, and 5. The rest of the populations (6–16) are in cluster 3. The DAPC analysis results thus confirm the barrier effect of the Marmara water body between Thrace and Anatolia on *A. chukar* individuals. On the other hand, the East and West populations have partially admixed clusters. While the populations within the Eastern cluster seem to be genetically similar, the populations in the Western cluster have a heterogeneous distribution. This regional pattern revealed by the DAPC analysis is also consistent with the results of the mtDNA sequences. The partial difference between the Eastern and Western populations indicates the existence of two major glacial refugia in Türkiye during the LGM. The demographic and spatial expansion of populations during the post-LGM period caused the admixture events between the East and West populations in some parts of Türkiye.

**Figure 5:**
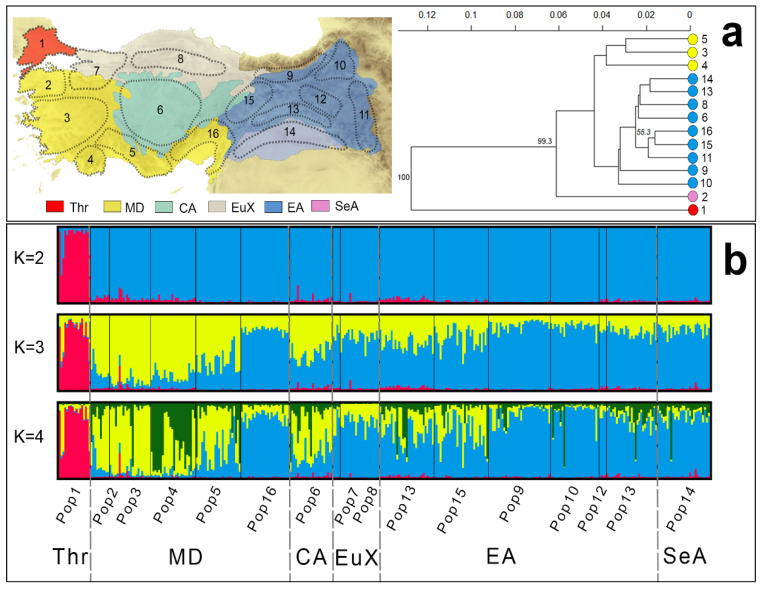
Genetic structure of *A. chukar* in Türkiye based on microsatellite analyses. The map shows the sampling areas and genetic clusters. (**a**) Nei distance dendrogram. (**b**) Structure analysis results for *A. chukar* genotypes computed by structure with K = 4. Each individual is represented as a vertical bar partitioned in K segments, whose length represents the assignment probability to the K = 4 cluster. Specimens are grouped according to their populations of origin.

The STRUCTURE analysis was conducted using the total sample set (347 individuals, 13 polymorphic microsatellite loci, 16 sampled populations) and K = 2–10. The Delta K test infers the population structure using the Bayesian analysis results, showing the maximum likelihood of the data when K = 4, the number of populations assumed (SMS5: Table S13). The estimated membership fractions of the populations for K = 2, 3, and 4 are presented in Figure 5b. The results of the STRUCTURE analysis indicate that population 1 differs from the other *A. chukar* populations, implying that the Marmara water body is a strong geographical barrier for the species in the West Palearctic. In K = 2, Thrace and the Anatolian mainland are represented by two different genetic clusters. In K = 3, the analysis suggests three genetic clusters (Thrace, East, and West) and a hybrid area throughout Anatolia, especially Central Anatolia. In K = 3, populations 2, 3, 4, and 5 represent the West genetic cluster, while populations 6–16 represent the East cluster, with the contact zone located in the area of populations 6, 13, and 15. However, in K = 4, in addition to the Thrace, East, and West genetic clusters, the China clade-B cluster appears within the East and West clusters. The microsatellite results indicate that clade-B contamination in Anatolia is approximately 12% (ranging from 2% to 54%). Intense contamination with China clade-B individuals was found in populations 4 (54%), 2 (20%), 6 (17%), and 11 (15%), whereas no contamination was detected in populations 1 and 8. The Nei distance dendrogram indicated that the Thrace, East, and West populations form three distinct genetic clusters (Figure 5a). In addition to these three clusters, population 2 forms a distinct genetic cluster separated from the Western cluster. Although population 16 is geographically located in the Mediterranean area in the dendrogram, it is genetically located in the Eastern cluster, close to population 15. Within the Eastern cluster, populations 9 and 10 are located in the basal branches of the dendrogram, indicating that these populations are genetically distinct from other members of the Eastern clusters.

### 3.6. ABC Method

The results of the ABC analysis, performed with 3 × 10^6^ simulations, indicated that hypothesis 2 was the most likely supported scenario (logistic regression posterior probability: 0.9830 (95% confidence intervals: 0.9802–0.9852), where the direct approach considered the closest 1000 points: 0.6260 (95% confidence intervals: 0.3261–0.9259)) (Figure 3, SMS6: Table S15). Hypothesis 2 predicted simultaneous divergence among the Thrace, East, and West populations at time t3, 49.140 yrBP (48.750–49.920 yrBP), with an admixture event between the East and West populations in Central Anatolia at time t1: 8.346 yrBP (7.371–13.026 yrBP) (Figure 3). Divergences among the Thrace, East, and West populations (ca. 50 kyBP) were dated to the middle of the Würm glacial period (ca. 115–117 kyBP). The admixture between the East and West lineages in Central Anatolia occurred in the post-LGM period, dated during or soon after the mini-ice age or Younger Dryas (ca. 12.9–11.7 kyBP). The gene flow from West to Central Anatolia was estimated to be 0.504, and that from East to West was estimated to be 0.496. This indicates almost identical gene flow ratios of the East and West populations to Central Anatolia.

### 3.7. Ecological Niche Modelling: Changing Geographic Distributions

**Figure 6:**
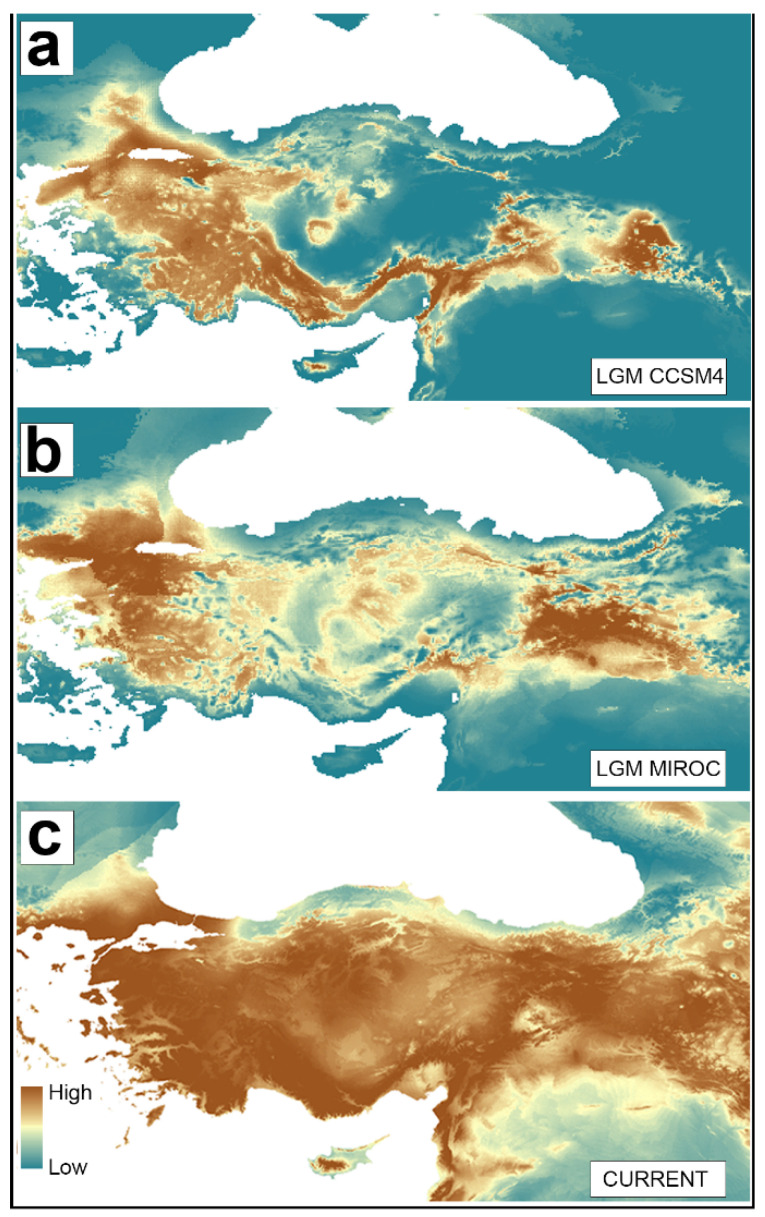
Predicted distributions of *A. chukar* based on ENMs using MaxEnt under bioclimatic conditions, predicted for the Last Glacial Maximum (LGM, ca. 22 kya) and the Present. (**a**) LGM-CSSM4 distribution model, (**b**) LGM-MIROC distribution model, and (**c**) Present distribution model. Warmer brown colors represent areas with higher bioclimatic suitability.

Ecological niche modelling (ENM) was used to determine whether the LGM distribution pattern of *A. chukar* in Türkiye was compatible with the results indicated by the two genetic markers (mtDNA and microsatellites). Considering the results of the ENMTOOLS analyses, we removed one of the bioclimatic layers that showed a high Pearson’s correlation coefficient value (≥80%) with the other layers. After removing the correlated bioclimatic layers, eight layers from CCSM and nine from MIROCH out of the 19 bioclimatic layers were retained as input data for the LGM, and nine were retained for the Present (SMS7: Table S16). The mean AUC values >0.9 for the *A. chukar* distribution models showed significance for all the binomial omission tests, indicating a good model performance (see SMS7: Table S16 for the AUC values and the contribution of the layers for each of the ENMs). All the distribution models for *A. chukar* populations in Türkiye in the LGM (Figure 6a,b) showed that the likelihood of suitability was low for the Black Sea coast and East Anatolian highlands. The LGM ENM results indicated that suitable conditions for *A. chukar* populations were only observed in the Mediterranean, West Anatolia, and the low-altitude and low-latitude areas of East Anatolia (Figure 6). 

In the LGM-CCSM model, southern Thrace, a large part of Western Anatolia, and the Mediterranean appear to be suitable areas for *A. chukar* distribution, whereas Central Anatolia, the Black Sea, and northern part of East Anatolia are not. The LGM-CCSM model also indicated that there was a suitable corridor for gene flow between the East and West populations via the Taurus Mountain belt. The LGM-MIROC model produced a result similar to that of the CCSM model, with some differences in the detailed distribution pattern. The model suggested that the distribution in the West and Mediterranean regions were concentrated in lowland areas, while low latitude was the major characteristic of the areas of *A. chukar* distribution in the East. However, the model also suggested some lowland micro-refugia in the northern part of the East Anatolia area, unlike the CCSM model. The MIROC model also showed several suitable areas for *A. chukar* distribution in Central Anatolia, although it did not support any connection between the East and West populations by way of the Mediterranean or Central Anatolian highlands (Figure 6). The current distribution model indicated that, except for the Black Sea coast, the *A. chukar* population is distributed throughout Türkiye from East to West. Comparing the ENM results for the LGM and the Present, the past and present distribution patterns seem to agree with the scenario suggested by the genetic data. Suitable habitat conditions, especially during the LGM, were absent in Central Anatolia and at high altitudes and latitudes in East Anatolia. This finding indicates that these areas could serve as barriers for *A. chukar* populations and suggests that the Thracian population’s divergence from populations in the other parts of Türkiye (or the Anatolian mainland) was not related to ecological conditions; rather, it resulted from fluctuations in the water levels of the Marmara water body during the LGM.

## 4. Discussion

### 4.1. Marmara Waterway and Its Barrier Effect on A. chukar

Both the mtDNA and microsatellite data results showed that *A. chukar* populations exhibit a genetic pattern that is well-structured in line with the geographic and topographic structure of Türkiye. The genetic structure of *A. chukar* in Türkiye supports three refugia or diversity areas: Thrace, East, and West. The differences between the Thracian and Anatolian mainland populations indicate that the Marmara waterway (also called the Turkish Straits System) is a barrier that prevents gene flow between *A. chukar* populations. Recent genetic studies also support the claim that the Marmara waterway limits the dispersal capability of many terrestrial organisms, including mammals, insects, and plants, between Anatolia and Thrace/the Balkans [40,51,135,136,137]. Because of differences in tectonic evolution and the complex climatic conditions of the Çanakkale and İstanbul Strait, the barrier effects and permeability of the two straits have also changed over time. The barrier effect of the Çanakkale Strait and its surrounding water channels in southern Thrace may have been maintained for the last 2.58 ky [138,139]. In contrast, the İstanbul Strait, formed by tectonic activities along the North Anatolian Fault north of the Marmara basin, is a relatively new formation (no older than ~100 ky) [140]. Before its formation, the barrier effect between Thrace and Anatolia north of Marmara was ensured by the İzmit–Sapanca/Adapazarı–Karasu corridors (Sakarya Bosphorus) during the Quaternary (perhaps for the last time during the Middle/Late Pleistocene) [141,142]. The barrier effect of the İzmit–Sapanca/Adapazarı–Karasu corridors is also supported by the presence of hybrid zones in the area [143,144]. There is disagreement regarding the Sea of Marmara’s water level during the LGM and its connection to the Black Sea via the İstanbul Strait.

Stratigraphic studies of the İstanbul Strait basin have indicated the presence of a freshwater lake system in the İstanbul Strait between 26.000 and 13.800 yrBP, when global sea levels were at their lowest [145,146]. This indicates that, during the LGM, the İstanbul Strait basin resembled a deep valley with a shallow lake–river system. Thus, it may have been a permeable barrier for *A. chukar* individuals. In the post-LGM period, however, the melting of glaciers in the northern latitudes caused a rapid rise in water levels in the Black Sea basin (~13.800 yrBP). The resulting catastrophic water flow from the Black Sea into the Marmara Sea raised the water level of the İstanbul Strait and Marmara Sea [147]. By ~12.000 yrBP, the rise in global sea levels had caused the present dual flow regime of the Çanakkale Strait, while the same dual flow pattern started at ~5.000 yrBP in the case of the İstanbul Strait [145]. 

Due to the euxinic climatic structure of the northern Marmara region, with high annual precipitation, the amount of water carried by the İstanbul Strait in the LGM may have been higher than previously thought. This implies that the İstanbul Strait’s barrier effect may have persisted during the LGM period, rather than the strait being a completely dry, deep valley. Depending on global sea level fluctuations throughout the Quaternary, the barrier effect of the Marmara waterway between Anatolia and Thrace/the Balkans followed a variable regime [148]. This may have played an important role in the differentiation of *A. greaca*–*A. chukar* and *A. chukar* (Anatolia)–*A. chukar* (Thrace) in the Western Palearctic.

The ENM analysis indicated that the climatic conditions of the northern Marmara region during the LGM were unsuitable for the distribution of *A. chukar* populations. Climatic conditions on the northern side of the Marmara waterway also created an ecological barrier during this period. In contrast, the ENM analysis showed that the southern part of Thrace could have been an important corridor for *A. chukar* distribution during the LGM. Since the Çanakkale Strait’s sea level was largely preserved during the LGM (−83 ± 2 m) [141], it could maintain its barrier effect. Nevertheless, global sea level fluctuations and the region’s complex tectonic evolution make it difficult to predict whether the Marmara waterway had a strong or soft barrier effect on *A. chukar* populations in the Quaternary.

The ABC simulation suggested that the Anatolia–Thrace/Balkans differentiation of the *A. chukar* populations occurred ~12.600 (12.500–12.800) generations ago. Assuming a generation time for *A. chukar* of 3.9 years, it was dated to 49.140 yrBP (48.750–49.920 yrBP). This date falls within the Würm glacial period (115–11.7 kya), when temperature fluctuations occurred. In particular, the last period, 57–29 kyBP, is quite complex in terms of changes in global sea levels [149,150]. During the Würm glacial period, there was a colder period during approximately 45–60 kyBP, when the Marmara Sea water level was lower than it is now (~−80 m) [139,142]. Therefore, its barrier effect between Anatolia and Thrace/the Balkans could have weakened during this period. Since *A. chukar* individuals do not fly well, the Marmara waterway and the straits system may have had an intermit barrier effect, depending on Quaternary climatic fluctuations, global sea level changes, and regional tectonics.

### 4.2. East and West Differentiation of A. chukar Populations in Türkiye

The main evolutionary factors shaping the present distribution of *A. chukar* populations in Türkiye are Türkiye’s heterogeneous topography, different climate types, and past climatic fluctuations. Although the long cooling periods experienced in the Late Pleistocene did not cause great glaciation in Türkiye, the ice ages affected the distribution and demography of many populations [36,65]. Today, Türkiye’s permanent snow layer begins at around 3.000–3.500 m elevation, while it may have begun at just 2.000 m at higher elevations during the LGM and 1.600 m in certain areas (the northern slopes) [13]. The distribution range of *A. chukar* populations during the LGM period may have shifted towards lower altitudes and southern latitudes in parallel with the cooling. The ENM analysis showed that the distribution of *A. chukar* populations during the LGM was restricted to low latitudes in East Türkiye, with the populations largely eradicated in the northeast regions. Both the MICRO and CCSM4 climate models showed that, during the LGML, there were no suitable habitats for *A. chukar* in the areas of population 9 and 10 in northeast Türkiye or that of population 11 in the east (Figure 6).

The eastern region of Türkiye is a high plateau with an average altitude above 2.000 m, which was mostly covered with glaciers and permafrost during the LGM [13]. These unsuitable ecological conditions limited the presence of *A. chukar* populations. In contrast, *A. chukar* populations remained widely distributed in west and south Türkiye due to the regions’ suitable climatic conditions (Figure 6). The demographic analyses also support this phenomenon by showing that the populations in eastern Türkiye were more severely affected by the cooling period than Western populations, and that contraction and expansion were more pronounced in the case of the Eastern populations (Table 1, SMS4: Figures S6 and S7). The CCSM climate model indicated that the Mediterranean coast of Türkiye, which is an important glacial refugium and biodiversity hotspot in the East Mediterranean Basin, was an important distribution area for *A. chukar* populations during the LGM (Table 1, Figure 6). The ENM analysis showed that southern Thrace and the areas around the Çanakkale Strait were also suitable distribution areas for *A. chukar* populations during the LGM (Figure 6). However, although the results indicated that climatic conditions were suitable in southern Thrace during the LGM, particularly in the Çanakkale Strait region, the demographic analyses of both the Cyt-b and D-loop regions did not support a significant expansion of populations 1 and 2. Moreover, the skyline plots indicate that the demographic structures of these two populations are stable (Table 1, SMS4: Figure S7). This may indicate the human-mediated genetic diversity erosion of these two populations. The Bayesian migration analysis results did not support any strong gene flow between populations 1 and 2 (SMS4: Table S10), which implies that the Çanakkale Strait maintained its barrier effect during the LGM. However, any human effects on the population structure of *A. chukar* in these areas may have erased the effects of past climatic and geologic events on these populations’ gene pools. The ENM analyses showed that there were no suitable areas for the distribution of *A. chukar* populations in the Anatolian mainland, throughout the Anatolian Diagonal, during the LGM (Figure 6). This indicates that the Anatolian Diagonal restricted gene flow between the East and West populations.

The ABC analysis estimated that the East–West and Thrace splits occurred simultaneously in the *A. chukar* populations approximately 12.500 generations ago (48.750 yrBP). This divergence time coincided with the end of a short warming period (45–60 kyBP) in the Würm glacial period [151]. This climatic period, which started at approximately 50 kyBP, affected the whole area and triggered the retreat of *A. chukar* populations to lower altitudes or southern latitudes in Türkiye (Figure 3c–f). According to the ABC analysis, after the fragmentation that started in the pre-LGM period (~50 kyaBP), the admixture event between the East and West populations in Central Anatolia occurred approximately 2.140 generations ago (8.346 yrBP) (Figure 3e). The mismatch analyses conducted on the Cyt-b gene produced similar results, indicating that the spatial and demographic expansion of *A. chukar* populations in Türkiye started at approximately 15 kyBP (8.3–22.7 kyBP) in parallel with post-LGM warming (Table 1). As a result of these demographic expansions, the hybridization of the East and West populations in Central Anatolia occurred at approximately 8 kyBP (6–13 kyBP), which coincided with the end of the mini-ice age or Younger Dryas (12.900–11.700 yrBP). Climatic warming during the post-LGM period decreased the barrier effect of the Anatolian Diagonal on *A. chukar* populations, thereby enabling gene flow between the Eastern and Western populations in Central Anatolia (Figure 3f). The ABC analysis indicates that the rates of gene flow from the East and West populations to Central Anatolia have almost the same ratio (ra_west_->CA and 1-ra_east_->CA: 0.5) (SMS5: Table S12). The Bayesian migration analyses performed on the Cyt-b data indicate that there was a weak gene flow bias between the East and West populations (SMS4: Table S10). That is, gene flow from East to Central Anatolia (60.7) was lower than that from West to Central Anatolia (115.4) (SMS4: Table S10). Similar results were obtained when the analysis was run for the Thrace, East, and West populations (M_east->west_: 66.1, M_west->east_: 176.6). This asymmetrical gene flow from west to east may be due to a rapid invasion of western *A. chukar* individuals, given the wider distribution range of western populations in the past and assortative mating between *A. chukar* individuals.

### 4.3. Conservation Genetics and Genetic Diversity

In this study, both mtDNA and nuDNA (microsatellites) indicated that *A. chukar* populations in Türkiye have high genetic diversity, except for all the genetic markers in population 1, which represents the Thracian part of Türkiye. In addition to the low haplotype and nucleotide diversity of this population, the haplotype distances within the population are very close to each other. This result implies that population 1 is affected by high inbreeding depression because of the founder effect, limited gene flow, or both. In addition, the samples from this region were mostly collected from Gökçe Island. Since islands generally provide limited habitat and food sources, island populations are very sensitive to changing environmental conditions and anthropogenic effects [152], making them more prone than mainland populations to entry into the vortex of extinction [153,154]. The genetic parameters also indicate that the Thrace population is genetically the most distinct population. In addition to a harsh environment, the founder effect and long-term isolation from the mainland accelerate the differentiation of island populations [155]. A few samples from the Thracian region were collected from Çanakkale, Edirne, and Tekirdağ Provinces in southern Thrace, where urbanization is low and the Mediterranean climate is dominant. In this respect, one of the important factors shaping Anatolia’s present distribution of *A. chukar* populations, in addition to, is human activities and domination in the area. The pressure of urbanization and the conversion of natural areas into agricultural lands are destroying the species’ natural habitats. To prevent human-mediated contamination, farm-reared *A. chukar* of unknown origin and *A. chukar* from other parts of Turkey should not be released in Thrace. Special conservation measures for the Thrace population are needed immediately. 

According to the Cyt-b results, the genetic diversity parameters of population 2 are higher than those of the other populations, with no China clade-B contaminations. This suggests that population 2 carries traces of old hybridization events between Anatolia and Thrace in its gene pool. Population 11 has the highest genetic diversity parameters in both mitochondrial markers. Population 11 is rooted in a special location, namely, the entrance to Anatolia from Asia. The high distance value between haplotypes in this population indicates that these areas may be another cryptic hybrid zone between Anatolian and Iranian *A. chukar* individuals. The microsatellite analyses indicated that the highest heterozygosity was observed in populations 16, 13, and 10. This may be due to their locations, as all three populations are situated in the intersection zones of different ecoregions [156]. Population 16 is biogeographically interesting, as it is located at the southern end of the Anatolian Diagonal and the intersection point of the east, southeast, and Mediterranean parts of Anatolia and the Levant. The area is a special part of the Anatolian biogeography and an important biodiversity hotspot region in the Eastern Mediterranean Basin.

### 4.4. Partridges in Türkiye and Congeneric Contaminations

Six of the seven species in the genus *Alectoris* are located in the Mediterranean Basin. The phylogeny of the genus also indicates that the Mediterranean Basin is the ancestral and diversification area for this genus [157]. As an important biodiversity and refugial area in the Eastern Mediterranean, Anatolia has played a central role in the evolution of both the genus and *A. chukar* [78,79]. Therefore, the question of whether the different species of the genus have a natural distribution in Türkiye is another controversial issue, especially considering *A. greaca* [31,158,159]. In the historical period, Anatolia played a crucial role in human migration and trade in the Mediterranean. This bridging effect renders Anatolia a possible core area of human-mediated genetic contamination of many species. *A. chukar* is one of the natural species endangered by human-mediated congeneric gene contamination. According to some studies, two species (*A. greaca* and *A. chukar*) are distributed in Anatolia [158,159,160]. They also reported that *A. greaca* is found in Western Anatolia, the coastal areas of Thrace and the Aegean region, and the Western Mediterranean. However, other studies concluded that only one species (*A. chukar*) is present in Türkiye, and that the natural distribution of *A. greaca* ends in the eastern part of the Balkans [79,161,162]. The morphological similarity of *A. chukar* and *A. greaca* undermines the reliability of phenotype-based diagnosis for these species. On the other hand, the Balkans and Western Thrace are the natural distribution areas of *A. greaca* and the areas with a high *A. chukar* × *A. greaca* hybridization probability. Both the mtDNA (Cyt-b and D-loop) and microsatellite results showed that Türkiye hosts no species of *Alectoris* other than *A. chukar*, with no congeneric contamination.

Based on morphological characteristics, authors of previous studies suggested that three subspecies of *A. chukar* are distributed in Türkiye: *A. c. cypriotes* Hartert, 1917, in the Mediterranean part of southern Türkiye and the Levant; *A. c. kleini* Hartert, 1925, in most of Anatolia north of the Taurus Mountains; and *A. c. kurdestanica* Meinertzhagen, 1923, at elevations extending from the southern Aras to the Zagros Mountains in the eastern part of Eastern Anatolia [79]. However, our results do not indicate the taxonomic status of these three subspecies.

### 4.5. Human-Mediated Artificial Hybridization

Artificial hybridizations caused by human activities can endanger many natural populations [24,163,164,165]. The uncontrolled release of species into natural areas may have many negative effects on their natural relatives, including deterioration of the genetic purity of local populations, reduced reproductive success, anomalies in embryonic development, reduced disease resistance, a weakened ability to obtain food, increased vulnerability to predation, and lower tolerance to physiological stress [166,167,168,169,170,171]. Natural populations of the most trafficked organisms are particularly affected by artificial hybridizations due to human activities [172,173,174,175]. Members of the Galliformes are the most affected by genetic contamination caused by human intervention [176,177,178]. The genus *Alectoris* may also face human-mediated artificial hybridization, because their economic importance and use in hunting have led to their introduction into different areas via trade routes [24,179,180]. Genetic contaminations observed in *Alectoris* species in Europe, where human-mediated artificial hybridizations are common, mostly stem from the China clade-B of *A. chukar* [30,31,77,80,181]. Such contamination can also be seen in *A. rufa* and *A. greaca* in Europe, which appears to be related to *A. chukar*, previously introduced into the Mediterranean Basin via the Silk Road [24,30,78,79,178]. Subsequently, the breeding of *A. chukar*-contaminated individuals with farm-reared chukars for hunting purposes contaminated the local gene pools of *A. rufa* and *A. greaca* in Europe, with *A. chukar* genes at a dangerous level [182,183,184,185].

The presence of China clade-B contamination in Türkiye was reported in a previous study [79]. However, this study was conducted on only a few individuals, which did not reflect the current levels of *A. chukar* contamination with China clade-B in Türkiye. Nevertheless, our results also support the presence of China clade-B contamination in Türkiye. The ratio of China clade-B contaminations in Türkiye is approximately 8% in mtDNA and about 12% in nuDNA. The absence of China clade-B contamination in Thrace is important in terms of the preservation of the genetic specificity and autonomy of this population. Among the contaminated regions, the highest contamination was observed in population 4 (44% mtDNA and 54% nuDNA). This may be because the major city in this population’s region, Antalya, is a historically important port settlement, which has served as an important trade area since ancient times. Alternatively, these contaminants may have originated from farm-reared *A. chukar* stocks, as in the European cases.

## 5. Conclusions

Three factors have played important roles in the formation of the genetic pattern of *A. chukar* in Türkiye: Türkiye’s geography–topography, past climatic fluctuations, and human-mediated artificial hybridizations. Traces of the influences of these three factors are clearly seen in the gene pool of *A. chukar* populations in Türkiye. The genetic structure is shaped in two ways: along an east–west cline due to the Anatolian Diagonal and a pattern with a distinct population in Thrace due to the barrier effects of the Marmara waterway. A contact/hybrid zone between the Eastern and Western clades arose in Central Anatolia. This contact zone formed around an area known as the Anatolian Diagonal due to the loss of its barrier effect during post-LGM warming. Interestingly, this hybrid zone is one of the largest clearly defined hybrid zones among all the Mediterranean refugia. Pleistocene climatic fluctuations had different effects on populations in the eastern and western parts of Türkiye. In particular, the West Anatolian populations were less affected by glaciation than those in the east. These results highlight the presence of cryptic micro-refugial areas in the eastern part that played an important role in preserving the lineages in the area during glaciation periods. While our results support the presence of China clade-B contamination in Türkiye, we found no evidence of *A. graeca* and no congeneric genetic contamination. Regarding the conservation of the species, the genetic differences between the Thrace, Western, and Eastern Anatolian populations should be considered, and only genetically controlled *A. chukar* stocks should be released into nature.

## Figures and Tables

**Table 1:** Cyt-b-based historical demographic indices of 14 populations and demographic expansion time estimations for 9 populations based on tau values.

Pop	N	Hs	Neutrality Tests		*g*	Mismatch Distribution					t (kyBP)
			Tajima’s D	Fu’s Fs		ϴ_0_	ϴ_1_	τ	SSD	HRI	
1	16	3	−0.57783	−0.50535	6242.766	0.113	6822.184	0.5	0.01030 ***	0.16333 ***	-
2	9	7	−0.32331	−3.22374 **	564.9102	0	6834.957	2.5	0.00355	0.05864	22.67
3 *	20	11	−1.0241	−4.12125 **	755.0651	0.61875	12.08988	2.05469	0.00213	0.02454	-
3	18	9	−0.92645	−4.40912 ***	981.1044	0	3464.392	1.875	0.003175	0.04916	17.00
4 *	22	11	−0.18624	−3.26323 *	847.2788	0	7.0633	5.87501	0.04654	0.10243	-
4	14	7	−1.05986	−3.43610 ***	956.1929	0	3438.142	1.5	0.01871	0.10361	13.60
5	22	9	−1.10051	−4.41119 **	381.5137	0.1125	3426.892	1.5	0.00974 *	0.10049 **	13.60
6 *	20	7	−0.60133	−1.43909	697.0333	0.16524	5.2273	2.08789	0.02734	0.10665	-
6	19	6	−0.97353	−0.91567	499.5347	0.009	6.197	1.855	0.0398	0.1466	-
8	20	10	−0.85245	−3.05118 *	888.0236	0.06327	6.73827	2.79689	0.00153	0.01864	-
9	20	10	−1.85148 *	−6.86623 ***	14083.83	0.225	3418.142	1.375	0.01153	0.11493	12.47
10 *	20	10	−1.33709	−2.64317	779.5263	2.54538	33.92714	1.1757	0.00275	0.01327	-
10	19	9	−1.07741	−2.11108	1650.156	2.14806	3812.59	0.98435	0.00233	0.01795	-
11 *	20	11	−1.02907	−4.16876 *	1211.932	1.12503	12.45129	2.2695	0.00257	0.01665	-
11	18	9	−1.09893	−3.22890 *	1893.151	0.0738	14.88743	2.46097	0.00205	0.02875	
13 *	20	9	−0.51238	−2.198	1434.16	0	4.77661	4.60548	0.01691	0.04078	-
13	15	7	−1.37083	−3.22307 **	1689.166	0	3418.142	1.375	0.00231	0.06077	12.46
14 *	21	11	−1.24432	−4.46319 **	2455.504	0.01406	9.08618	3.28711	0.00324	0.01823	
14	18	10	−1.46447	−5.32583 ***	2335.765	0	28.3962	2.13281	0.00198	0.0355	19.34
15	20	10	−0.91599	−5.64103 ***	4217.226	0.03692	11.93892	1.74023	0.00947	0.06194	15.78
16	20	7	−1.77344 *	−3.29864 **	10306.32	0.73653	3444.476	0.89680	0.01642	0.10618	8.13

Explanations: The genetic diversity indices for the populations, from top to bottom, are Watterson Theta (θ). Based on a model of sudden population expansion, Tajima’s D and Fu’s Fs are expected to be significantly negative. Mismatch analysis parameters: θ_0_ and θ_1_ are the substitution rates before and after the expansion, respectively. Expansion parameter τ (with lower and upper bounds at α = 0.05). SSD tests the validity of a stepwise expansion model based on the sum of squares deviations between the observed and expected mismatch with probability values (P). Non-significant mismatch values suggest population expansion. Harpendin’s raggedness index is calculated similarly, with probability values (P). Non-significant mismatch values suggest population expansion. Time since lineage expansion (t) is calculated from τ = 2 µt, where µ = 0.21%. The MYR was 1024 bp, while the generation time was taken to be 3.9 years.

## Data Availability

The DNA sequences are deposited in GenBank, and the accession numbers are added to Supplementary Material S2. Details regarding individual samples are available in Table S3.

## Supplementary Materials

The following supporting information can be downloaded at: https://www.mdpi.com/article/10.3390/biology12030401/s1, Supplementary Materials S1: Sample locations. Supplementary Material S2: Labor-atory work, primers, GenBank accession numbers, and haplotype frequency. Supplementary Material S3: Phylogeny. Supplementary Material S4: Population genetics. Supplementary Material S5: Microsatellites. Supplementary Material S6: DIYABC. Supplementary Material S7: ENM. [128,129,186,187,188].
