# Supplementary material for: Geographic Genetic Structure of Alectoris chukar in Türkiye: Post-LGM-Induced Hybridization and Human-Mediated Contaminations
Source: Biology (Basel). 2023 Mar 3;12(3):401. doi: 10.3390/biology12030401 (PMC10045126; doi:10.3390/biology12030401)
Supplement: Supplementary file 1 [file biology-12-00401-s001.zip › 1 - Supplementary Material S1 - Sample locations.pdf]

Geographic genetic structure of *A. chukar* in Türkiye: Post-LGM induced hybridization and human-mediated contaminations

Sarp Kaya, Bekir KABASAKAL, Ali ERDOĞAN

Supplementary information S1: Sampling Localities

While a few samples were obtained from population 7 (4 samples) and population 12 (7 samples), the highest number of samples were collected from population 13 (26 samples). We collected samples in Manisa (population 3) at 36 m the lowest altitude and in Ardahan (population 10) at 2177 m, which is the highest altitude within the distribution range of chukar partridges. In the sample areas, we detected the lowest average elevation at 197 m in population 1, and the highest at 1936 m in population 10.

Table S1. Sampling details (see Fig. 1) of *Alectoris chukar* specimens from Türkiye, these samples were used for all the analysis (Reg.: Region Pop: population number)

Reg.	Pop.	Location	Latitude	Longitude	Elevation (m)	Date	Num. of samples	Total
1	1	Edirne, Pınar	40.686016°	26.606369°	195	2.08.2017	1	20
	2	Çanakkale, Gökçeada	40.141424°	25.749488°	180	24.12.2017	16	
	3	Çanakkale, Gelibolu	40.375558°	26.578530°	190	11.11.2017	2	
	4	Tekirdağ, Şarköy	40.629267°	27.065405°	147	15.04.2018	1	
2	1	Çanakkale, Kirazlı Bucağı	40.034714°	26.697445°	352	4.11.2017	5	20
	2	Balıkesir, Marmara Adası	40.619383°	27.619148°	476	20.07.2017	1	
	3	Balıkesir, Çamköy	39.526365°	27.739588°	355	11.08.2017	2	
	4	Çanakkale, Biga road	40.203184°	27.206451°	191	8.04.2018	12	
3	1	Afyonkarahisar, Şuhut	38.536621°	30.524428°	1183	5.11.2017	2	20
	2	Afyonkarahisar, İncesu	38.110355°	30.330945°	1626	24.09.2017	3	
	3	İzmir, Torbalı	38.199293°	27.388455°	270	7.09.2017	3	
	4	İzmir, Urla	38.312066°	26.598190°	333	21.10.2017	3	
	5	Akhisar, Kavaklanı State Hunting Ground	38.884125°	27.896218°	226	7.01.2018	2	
	6	Manisa, Saruhanlı	38.749163°	27.543427°	36	9.12.2017	2	
	7	Manisa, Salihli	38.467846°	28.138202°	227	9.12.2017	2	
	8	Denizli, Pamukkale	37.901485°	29.171536°	363	5.11.2017	1	
	9	İzmir, Menderes	38.64482°	27.153969°	280	12.05.2018	2	
4	1	Antalya, Korkuteli	37.091590°	30.378576°	869	20.07.2017	8	20
	2	Antalya, Elmalı	36.778389°	29.949287°	1330	30.03.2017	6	
	3	Antalya, Kaş	36.210390°	29.648247°	470	13.04.2017	3	
	4	Muğla, Fethiye, Seki	36.794206°	29.682371°	1705	17.06.2017	1	

Reg.	Pop.	Location	Latitude	Longitude	Elevation (m)	Date	Num. of samples	Total
	5	Burdur, Karamanlı	37.352578°	29.810414°	1109	28.10.2017	2	
5	1	Burdur Karaçal	37.561920°	30.040920°	937	28.10.2017	4	20
	2	Burdur, Ağlasun State Hunting Ground	37.616995°	30.565215°	1275	28.10.2017	2	
	3	Isparta, Senirkent	38.099140°	30.557642°	1145	28.10.2017	5	
	4	Mersin, Mut	36.578316°	33.293603°	260	8.11.2017	4	
	5	Karaman, Ayrancı	37.119382°	33.228724°	1168	19.11.2017	1	
	6	Karaman, Akpınar	37.028138°	33.418603°	1347	4.11.2017	1	
	7	Mersin Mut, Adras State Hunting Ground	36.383941°	33.111137°	655	22.05.2018	3	
6	1	Kırşehir, Çiçek Dağı	39.579880°	34.309097°	1317	18.05.2017	3	22
	2	Kırşehir, Kaman	39.243477°	33.975380°	1522	21.07.2017	4	
	3	Ankara, Nallıhan	40.193449°	31.330837°	829	30.07.2017	11	
	4	Eskişehir, Mihalcik	39.835875°	31.774095°	768	28.03.2018	2	
	5	Ankara, Bala	39.568236°	33.142025°	1274	16.09.2017	2	
7	1	Bilecik, İnhisar State Hunting Ground	40.036137°	30.375168°	286	23.07.2017	2	4
	2	Bursa, Gemlik	40.172722°	29.055880°	585	11.08.2017	2	
8	1	Çorum, Merkez	40.554426°	35.017798°	1181	24.07.2017	8	20
	2	Çankırı, Çerkeş	40.957594°	32.959572°	1573	24.03.2018	8	
	3	Çankırı, Kurşunlu	40.867550°	33.251540°	1284	24.03.2018	2	
	4	Çorum, Örencik köyü	40.372254°	35.181812°	734	25.03.2018	1	
	5	Çorum, , Mecitözü	40.492845°	35.274091°	929	25.03.2018	1	
9	1	Bayburt, Aydıntepe	40.380617°	40.160147°	1577	10.08.2017	4	20
	2	Erzincan, Çayırlı	39.829291°	39.958642°	1596	26.05.2017	3	
	3	Gümüşhane, Çevrepınar	40.270369°	39.277054°	2130	6.08.2107	1	
	4	Bayburt, Karşıgeçit	40.399424°	40.408837°	1555	26.03.2018	5	
	5	Bayburt, Gökçeeli	40.434522°	40.347470°	1989	26.03.2018	4	
	6	Bayburt, Aslandede	40.222072°	40.273755°	1560	27.03.2018	3	
10	1	Ardahan, Çıldır	41.108409°	42.792463°	2177	14.11.2017	1	20
	2	Erzurum, Tortum	40.319294°	41.561724°	1774	4.11.2017	13	
	3	Kars, Kağızman	40.104822°	43.098657°	1857	9.08.2017	6	
11	1	Ağrı, Doğubeyazıt	39.526540°	44.107937°	1695	15.11.2017	3	21
	2	Van, Gevaş	38.301213°	42.971331°	1760	20.07.2017	3	
	3	Van, Erciş	39.106891°	43.326325°	1724	20.07.2017	3	
	4	Hakkari, Bağışlı	37.739725°	44.038866°	2115	24.07.2017	6	
	5	Iğdır, Tuzluca	40.084180°	43.734326°	939	27.07.2017	6	
12	1	Bingöl, Derinçay	39.128705°	40.789506°	1705	4.08.2017	2	7
	2	Bitlis, Ahlat	38.776040°	42.466048°	1854	26.07.2017	5	
13	1	Adıyaman, Kahta-Bağlar	37.769586°	38.590981°	674	1.08.2017	3	26

Reg.	Pop.	Location	Latitude	Longitude	Elevation (m)	Date	Num. of samples	Total
	2	Adıyaman, Gerger	38.023461°	39.033642°	705	4.08.2017	2	
	3	Diyarbakır, Çürgüş	38.273231°	39.323364°	1500	2.08.2017	17	
	4	Batman, Sasan	38.338341°	41.431257°	1019	26.07.2017	3	
	5	Siirt, merkez	37.886719°	41.974993°	793	24.07.2017	1	
**14**	1	Gaziantep, Geneyik,	36.920356°	37.325668°	790	16.08.2017	10	**20**
	2	Mardin, Yaylacık	37.426836°	40.700483°	1040	20.07.2017	5	
	3	Şırnak, Çakırsöğüt	37.534889°	42.420039°	1226	24.07.2017	5	
**15**	1	Malatya, Darılı	38.916255°	37.612359°	1338	1.08.2017	11	**22**
	2	Elazığ, Akuşağı	38.710963°	38.575805°	1011	2.08.2017	1	
	3	Tunceli, Muzur Vadisi	39.277444°	39.367797°	1476	20.07.2017	6	
	4	Sivas, Divriği,	39.178189°	38.275369°	1191	9.08.2017	4	
**16**	1	Hatay, Belen	36.489822°	36.169606°	609	10.08.2017	1	**24**
	2	Kahramanmaraş, Dereli	37.616667°	36.922549°	1058	3.05.2017	10	
	3	Kayseri, Yahyal	38.051461°	35.497958°	1551	17.05.2017	10	
	4	Niğde, Aladağlar	37.735580°	35.037009°	1665	6.08.2017	2	
	5	Adana, Pozantı	37.453000°	34.898075°	878	10.08.2017	1	
