# Supplementary material for: Geographic Genetic Structure of Alectoris chukar in Türkiye: Post-LGM-Induced Hybridization and Human-Mediated Contaminations
Source: Biology (Basel). 2023 Mar 3;12(3):401. doi: 10.3390/biology12030401 (PMC10045126; doi:10.3390/biology12030401)
Supplement: Supplementary file 1 [file biology-12-00401-s001.zip › 2 - Supplementary Material S2 - Laboratory work, primers, GenBank accession numbers and haplotype frequency.pdf]

Geographic genetic structure of A. chukar in Türkiye: Post-LGM induced hybridization and human-mediated contaminations

Sarp KAYA, Bekir KABASAKAL, Ali ERDOĞAN

Supplementary information S2: Laboratory work, primers, GenBank accession numbers and haplotype frequency

Mt-DNA Amplification and Sequencing

A 1092 bp region of the Cyt-b region and a 1115 bp D-loop region of Mt-DNA were amplified by PCR reaction using the universal primers given in Table SI1. Total DNA was extracted from 300 specimens preserved in 96 % ethanol kept at -20 °C (SI1 Table S1) with GeneJET Genomic DNA Purification Kit (Thermo Scientific #K0722) following the manufacturer's instructions. Amplifications were performed in a 50 µl volume containing 1 µl of each primer (10 µM), 1 µl dNTPs mix (10 mM), 3.2 µl 25 mM $MgCl_2$, 5 µl 10X Platinum PCR buffer (containing 200 mM Tris–HCl [pH 8.4], 500 mM KCl), 0.5 U Platinium Taq DNA polymerase (Invitrogen), and 20-50ng template DNA. PCR was carried out in an Applied Biosystems SimpliAmp Thermal Cycler. Cycling conditions for both Mt-DNA regions were 30 sec denaturing at 95 °C; (20 sec at 95 °C, 30 sec at 41 °C, and 1.10 sec at 72 °C) x 25 cycles. PCR products were purified and then sequenced in both directions on an ABI373 automated sequencer by Macrogen (Macrogen Inc., Amsterdam, Netherlands). Sequences were deposited in GenBank and the Accession Numbers were given in Table S2.

Microsatellite genotyping

A total of 17 autosomal loci that were originally developed for chicken (*Gallus gallus*) were used for genotyping of *A. chukar* populations. Since no variation was detected in 4 (MCW0104, MCW152, Aru1.22 and MCW0301) of the 17 loci, analyses were performed only with the 13 polymorphic loci. All forward primers were synthesised with fluorescently labelled (Table S1). DNA was amplified in 15µl reaction volume containing 1µl $MgCl_2$ (25mM), 1µl dNTPs mix (10mM), 0.6µl each primer pair (forward and reverse- 10µM) and 0.5 U Taq DNA polymerase. Thermal cycling was carried out in an Applied Biosystems SimpliAmp Thermal Cycler. Cycling conditions were 30 sec denaturing at 95 °C; (20 sec at 95 °C, 30 sec at 50 °C, 55 °C, 57 °C, 61 °C, 65 °C for each loci used different annealing temperatures see Table SI1, and 60 sec at 72 °C for extension) x 30 for each PCR reaction separately.

For fragment analysis, we multiplexed PCR products in a single 96-well plate (MCW118/MCW225/MCW280/MCW0069, Aru1.22/Aru1E7, MCW0121/Aru1.23/Aru1.27/MCW146, and MCW135, MCW276, MCW0301). Each multiplexed set was arranged according to the type of fluorescent label and the length of the markers. Fragment analyses were performed using 500 Liz size standards on ABI 3730xl 96-cacapillaryNA analyser by Macrogen (Macrogen Inc., Amsterdam, Netherlands).

Table S2. Primer and annealing information of two Mt-DNA regions (D-loop and Cyt-b) and seventeen microsatellite markers were used in this study.

**Mt-DNA**					
**No**	**Locus**	**Length (bp)**	**Forward**	**Reverse**	**Annealing °C**
1	D-loop	1155	AGGACTACGGCTTGAAAAGC	TAGTAAGGTTAGGACTAAGTCTT	41
2	Cyt-b	1092	ATGGCACCCAATATCCGAAAATC	TTCGTAGTTGAGGATTTTATTTTGAAG	41

**Microsatellite markers (Nu-DNA)**							
**No**	**Locus**	**Repeat Motif**	**Allele range (bp)**	**Forward**	**Reverse**	**Annealing °C**	**Fluorescent Dye (5' forward ends)**
1	MCW0104	(TG)9	100-150	TAGCACAACTCA AGCTGTGAG	AGACTTGCAC AGCTGTGACC	57	Ned
2	MCW118	(TA)2(TAT G)3[TA(TA TG)2]2(TA) 33(TATG)	150-200	ATGATGAAGCAT TTAGTCTAAG	CAATTTACTCA GAGATGCAGT G	50	6-FAM
3	MCW0121	(4TG)7	200-250	ATGGATAGGGGT AACTGTTGC	CTACGTGTGTT TGACAGCTGG	61	Ned
4	MCW135	(CA)10	100-150	ATATGCTGCAGA GGGCAGTAG	CATGTTCTGCA TTATTGCTCC	55	6-FAM
5	MCW225	(TG)13	150-200	AACGGACTCTTC TGTCTATAG	TGCTTTGCTCC TCATTAAAGG	50	Vic
6	MCW276	(TG)4	200-250	ACTCTGAGTGGA ATTACCT	TTTCTGTTAGA AGCAGCTGC	55	Vic
7	MCW280	(AT)8	200-250	TGAATGGTTTTA TGCATTGT	AGCAACATATC CATAAGTGT	50	Ned
8	MCW152	(AAAC)6	200-250	GAGGTATTTCTC AGAACTTCC	CAAACTATTAG TTCTTCAGCTG	65	Ned

9	Aru1.22	(GT)9	220-270	ATGTGAGTGTGT AAGGGGGAGT	GACATCTGGC ACATAAAAAT CAAG	57	6-FAM
10	Aru1.23	(TG)16	200-250	GTAAACTTGCCC CCTGCTGTTC	CTTCTCTGGGC AGCTGTGTC	61	6-FAM
11	Aru1.27	(AT)14	150-200	GTTCTGGCTTTA AAGAGCTTGG	TGAGAATGCA GGACAGGAGA TA	61	Pet
12	Aru_1E97	(GACA)32	250-350	GCTGTGATTCCA TTGGAGAC	GGCGTATGTCT GCATGTAAG	57	Vic
13	MCW_146	(TG)11	130-180	CCGTGTGGTGAA CAACGATGA	CAAATCTGCCC TGACGTCAGC	61	Vic
14	Aru_1E7	(GGAT)12	200-250	CCACTTCACATC AACACCCA	GACACTGGATT TCGGTTTGG	57	Pet
15	Aru_1E45	(CA)22	100-200	TACAGCAGTGG AGGTGGTTG	ACTGCATTGTG GACCTTCTG	65	Pet
16	MCW0301	(TG)25	260-302	GGAGAGGAGAC AACTGTATTC	AGGGTGAGAG GTAACAAGTG C	55	6-FAM
17	MCW0069	(TG)11	200-250	GCACTCGAGAA AACTTCCTGCG	ATTGCTTCAGC AAGCATGGGA GGA	50	Pet

Table S3. GenBank accession numbers for each of D-loop and Cyt-b haplotypes.

D-loop						Cyt-b		
No	Hap. Abb.	Acc. No.	No	Hap. Abb.	Acc. No.	No	Hap. Abb.	Acc. No.
1	AfynD1	OQ472162	62	IgdrD2	OQ472247	1	AfynC	OQ472083
2	AfynD2	OQ472154	63	IsprtD1	OQ472143	2	AnkaraC	OQ472112
3	AgriD	OQ472206	64	IsprtD2	OQ472240	3	AntC1	OQ472137
4	AnkaraD1	OQ472156	65	IzmrD1	OQ472146	4	AntC2	OQ472135
5	AnkaraD2	OQ472170	66	IzmrD2	OQ472147	5	AntC3	OQ472134
6	AnkaraD3	OQ472176	67	IzmrD3	OQ472148	6	AntC4	OQ472133
7	AnkaraD4	OQ472177	68	IzmrD4	OQ472180	7	AntC5	OQ472091
8	AnkaraD5	OQ472178	69	KarmanD1	OQ472248	8	AntC6	OQ472117
9	AnkaraD6	OQ472188	70	KarsD1	OQ472163	9	AntepC	OQ472125
10	AnkaraD7	OQ472224	71	KarsD2	OQ472200	10	BalksrC1	OQ472107
11	AntD1	OQ472242	72	KarsD3	OQ472164	11	BalksrC2	OQ472116
12	AntD2	OQ472155	73	KarsD4	OQ472171	12	BatmanC	OQ472127
13	AntD3	OQ472149	74	KarsD5	OQ472192	13	BaybrtC1	OQ472093
14	AntD4	OQ472144	75	KarsD6	OQ472195	14	BaybrtC2	OQ472126
15	AntD5	OQ472175	76	KaysriD1	OQ472217	15	BinglC	OQ472105
16	AntD6	OQ472145	77	KaysriD2	OQ472181	16	BitlsC	OQ472103

17	AntD7	OQ472241	78	KaysriD3	OQ472182	17	BurdrC	OQ472108
18	AntD8	OQ472139	79	KrshrD	OQ472179	18	CankrC1	OQ472079
19	AntD9	OQ472165	80	MansaD1	OQ472168	19	CankrC2	OQ472080
20	AntepD1	OQ472157	81	MansaD2	OQ472173	20	CankrC3	OQ472082
21	AntepD2	OQ472159	82	MansaD3	OQ472189	21	CankrC4	OQ472099
22	AntepD3	OQ472244	83	MansaD4	OQ472238	22	CankrC5	OQ472131
23	AntepD4	OQ472230	84	MarasD1	OQ472221	23	CnakleC1	OQ472087
24	BalksrD1	OQ472186	85	MardnD1	OQ472158	24	CnakleC2	OQ472114
25	BalksrD2	OQ472187	86	MardnD2	OQ472160	25	CnakleC3	OQ472123
26	BatmanD1	OQ472219	87	MardnD3	OQ472193	26	CormC1	OQ472078
27	BaybrtD1	OQ472207	88	MersnD1	OQ472161	27	CormC2	OQ472081
28	BaybrtD2	OQ472208	89	MersnD2	OQ472174	28	ElazgC1	OQ472096
29	BaybrtD3	OQ472209	90	MersnD3	OQ472246	29	ElazgC2	OQ472119
30	BaybrtD4	OQ472211	91	MlatyaD1	OQ472218	30	ErzncnC	OQ472122
31	BileckD	OQ472215	92	MlatyaD2	OQ472150	31	ErzrmC1	OQ472090
32	BingolD	OQ472228	93	MlatyaD3	OQ472151	32	ErzrmC2	OQ472092
33	BurdrD1	OQ472142	94	MlatyaD4	OQ472152	33	EskshrC	OQ472110
34	BurdrD2	OQ472167	95	MuglaD1	OQ472140	34	GmshnC	OQ472128
35	BurdrD3	OQ472245	96	MuglaD2	OQ472141	35	IgdrC1	OQ472101
36	BurdrD4	OQ472250	97	MusD1	OQ472223	36	IgdrC2	OQ472102
37	BursaD1	OQ472214	98	MusD2	OQ472203	37	IsprtC1	OQ472106
38	BursaD2	OQ472216	99	NevshrD	OQ472212	38	IsprtC2	OQ472124
39	CankrD1	OQ472233	100	NigdeD1	OQ472183	39	IzmrC1	OQ472084
40	CnakleD1	OQ472185	101	SiirtD1	OQ472197	40	IzmrC2	OQ472111
41	CnakleD2	OQ472237	102	SiirtD2	OQ472234	41	IzmrC3	OQ472118
42	CnakleD3	OQ472190	103	SiirtD3	OQ472201	42	KarsC1	OQ472138
43	CnakleD4	OQ472191	104	SiirtD4	OQ472205	43	KarsC2	OQ472085
44	CnakleD5	OQ472253	105	SiirtD5	OQ472220	44	KarsC3	OQ472100
45	CnakleD6	OQ472243	106	SivasD	OQ472252	45	KarsC4	OQ472104
46	CnakleD7	OQ472196	107	TekrdgD	OQ472235	46	KarsC5	OQ472120
47	CormD1	OQ472199	108	TuncliD1	OQ472153	47	KrshrC1	OQ472132
48	CormD2	OQ472213	109	TuncliD2	OQ472172	48	KrshrC2	OQ472088
49	DenzliD1	OQ472210	110	TuncliD3	OQ472198	49	MansaC1	OQ472109
50	DyrbkrD1	OQ472231	111	TuncliD4	OQ472227	50	MansaC2	OQ472130
51	DyrbkrD2	OQ472229	112	UrfaD1	OQ472194	51	MarasC1	OQ472094
52	DyrbkrD3	OQ472204	113	UrfaD2	OQ472251	52	MarasC2	OQ472098
53	DyrbkrD4	OQ472232	114	VanD1	OQ472166	53	MardnC1	OQ472086
54	ElazgD1	OQ472225	115	VanD2	OQ472202	54	MardnC2	OQ472115
55	ElazgD2	OQ472226				55	MersnC1	OQ472089
56	ErzrmD	OQ472239				56	MersnC2	OQ472097
57	EskshrD1	OQ472236				57	MlatyaC	OQ472121
58	HakkriD1	OQ472184				58	MuglaC	OQ472129
59	HakkriD2	OQ472169				59	SivasC1	OQ472095
60	HatayD	OQ472249				60	UrfaC	OQ472113
61	IgdrD1	OQ472222				61	VanC	OQ472136

Tablo S4. The haplotype sharing table of 90 Cyt-b haplotypes was obtained from the study. The Cyt-b haplotypes used in the analyses from NCBI and this study.

Species	Haplotype	Regions																	GenBank (NCBI)
		1	2	3	4	5	6	7	8	9	10	11	12	13	14	15	16	Frq	
Tetraogallus himalayensis	NC_027279																	1	
Coturnix coturnix	L08377																	1	
A. melanocephala	Z48773																	1	
A. barbara	AM492953																	1	
A. rufa	AM850848																	1	
	AM850843																	1	
	AM850844																	1	
	AM850837																	1	
	AM85083																	1	
	AM850842																	1	
	AM850846																	1	
	AM850835																	1	
	AM850831																	1	
	AM850849																	1	
	AM850851																	1	
A. greaca	AM850717																	2	AM492952
	AM850716																	3	AM492951, FM203237
A. magna	EU839473																	1	
A. philbyi	Z48774																	1	
A. chukar-B	EU839475																	1	
A. chukar-B	AM850750																	1	

Species	Haplotype	Regions																Frq	GenBank (NCBI)
		**1**	**2**	**3**	**4**	**5**	**6**	**7**	**8**	**9**	**10**	**11**	**12**	**13**	**14**	**15**	**16**		
A. chukar-B	AM850737																	1	
A. chukar-B	AM850734																	1	
A. chukar-B	AM850729																	1	
A. chukar-B	AM850751																	1	
A. chukar falki-B	GU214293																	1	
A. chukar falki-B	GU214294																	1	
A. chukar pubescens -B	GU214292																	1	
A. chukar pubescens -B	GU214291																	1	
A. chukar-B	AntC1				1													1	-
	VanC											1						1	-
	AntC2				1													1	-
	KrshrC1						1											1	-
	AntC3				1										1			3	AM850755
	AntC4 **(D)**			2	5		1				1	1		4	3			19	AM850718, AM850786
A. chukar-A	AfynC			2														2	
	AnkaraC						1											1	
	AntC5				1													1	
	AntC6				1													1	
	AntepC														1			1	
	BalksrC1		1															1	
	BalksrC2		1															1	
	BatmanC													1				1	
	BaybrtC1									1								1	
	BaybrtC2									1					1			2	

Species	Haplotype	Regions																	GenBank (NCBI)
		1	2	3	4	5	6	7	8	9	10	11	12	13	14	15	16	Frq	
	BinglC **(B)**	2	3	1	7	8			2	1		2	1			1		28	
	BitlsC											1		1		1		3	
	BurdrC					1												1	
	CankrC1								1									1	
	CankrC2								1									1	
	CankrC3 **(A)**	12	1	7	1	7	6	3	8	9	6	5	2	8	8	9	12	104	
	CankrC4 **(H)**							1	1		2			1	1			6	
	CankrC5								1									1	
	CnakleC1	2																2	
	CnakleC2		2															2	
	CnakleC3		1															1	
	CormC1								1									1	
	CormC2								1									1	
	ElazgC1											1				1		2	
	ElazgC2 **(F)**										2	1		1	2	2	2	10	
	ErzncnC									1								1	
	ErzrmC1									1								1	
	ErzrmC2									1								1	
	EskshrC **(G)**		1			2	2								1	1		7	
	GmshnC									1								1	
	IgdrC1														1			1	
	IgdrC2										1	2						3	
	IsprtC1					1												1	
	IsprtC2					1												1	

**Species**	**Haplotype**	**Regions**																	**GenBank** (NCBI)
		**1**	**2**	**3**	**4**	**5**	**6**	**7**	**8**	**9**	**10**	**11**	**12**	**13**	**14**	**15**	**16**	**Frq**	
	IzmrC1			1														1	
	IzmrC2			1														1	
	IzmrC3			1														1	
	KarsC1										1							1	
	KarsC2										1							1	
	KarsC3 **(E)**								1	3	3			3	2	2	1	15	
	KarsC4										2	2		1				5	
	KarsC5										1							1	
	KrshrC2				1		1											2	
	MansaC1 **(C)**			3	1	1	8		2	1				1		1		18	
	MansaC2			1														1	
	MarasC1																1	1	
	MarasC2													1			1	2	
	MardnC1														1			1	
	MardnC2														1			1	
	MersnC1			1		3												4	
	MersnC2					1												1	
	MlatyaC															1		1	
	MuglaC				1													1	
	SivasC															1		1	
	UrfaC														1			1	

Tablo S5. The haplotype-sharing table of 142 D-loop haplotypes was obtained from the study. The D-loop haplotypes used in the analyses from NCBI and this study.

Species	Haplotype	Regions																	GenBank
		1	2	3	4	5	6	7	8	9	10	11	12	13	14	15	16	Frq	
Tetraogallus himalayensis	NC_027279																	1	
Coturnix japonica	NC_003408																	1	
A. melanocephala	AJ222736																	3	AJ222735, AJ222734
A. barbara	AM492998																	1	
	AJ222727																	1	
	AJ222726																	1	
A. philbyi	AJ222738																	1	
	AJ222737																	1	
	AJ005574																	1	
A. magna	AJ222733																	1	
	AJ222732																	1	
A. graeca	AM492997																	2	AM850717
	AM084673																	2	AM850716
A. rufa	AJ586197																	2	
	AJ586225																	1	
	AJ586192																	1	
	AJ586195																	1	
	AJ586194																	1	
	Y08555																	1	
A. chukar-B	AM850786																	1	
	AM850735																	2	AM850826
	AM850737																	1	

Species	Haplotype	Regions																	GenBank
		1	2	3	4	5	6	7	8	9	10	11	12	13	14	15	16	Frq	
	EF688280																	1	
	EF688282																	1	
	AM850750																	1	
	AJ586201																	1	
	VanD1											2			3			8	AM850718, AM850751 AM850730
	AntD1				4		1				1				4			10	AM850755
	AntD2				1													1	
	AfynD1			1														1	
A. chukar-A	AM850810					1								1				2	2
	AfynC2			1														1	
	AgriD									1		1						2	
	AnkaraD1						1											1	
	AnkaraD2						1											1	
	AnkaraD3						1											1	
	AnkaraD4						1											1	
	AnkaraD5						1											1	
	AnkaraD6						1											1	
	AnkaraD7						1											1	
	AntD3				1											1		2	
	AntD4				6													6	
	AntD5				1	2			1	1			2			1		8	
	AntD6				2													2	
	AntD7			6	1	3	3		4	3		2				1	6	29	
	AntD8				1													1	

Species	Haplotype	Regions																	GenBank
		1	2	3	4	5	6	7	8	9	10	11	12	13	14	15	16	Frq	
	AntD9				1													1	
	AntepD1														1			1	
	AntepD2														1			1	
	AntepD3	1				1	1								1			4	
	AntepD4													2	2	1	1	6	
	BalksrD1		1															1	
	BalksrD2		1															1	
	BatmanD1													1				1	
	BaybrtD1									1								1	
	BaybrtD2									1								1	
	BaybrtD3									2								2	
	BaybrtD4									1								1	
	BileckD							1										1	
	BingolD												1					1	
	BurdrD1					1												1	
	BurdrD2					3		1	3			1						8	
	BurdrD3					1												1	
	BurdrD4					5												1	
	BursaD1							1										1	
	BursaD2							1										1	
	CankrD			1					1		3	1					1	7	
	CnakleD1		1															1	
	CnakleD2	1					1											2	
	CnakleD3	2																2	
	CnakleD4		3															3	
	CnakleD5	2									1			2				5	
	CnakleD6	7	3				1											11	

Species	Haplotype	Regions																	GenBank
		1	2	3	4	5	6	7	8	9	10	11	12	13	14	15	16	Frq	
	CnakleD7		1															1	
	CormD1								3									3	
	CormD2								1									1	
	DenzliD			1		1												2	
	DyrbkrD1						1		3					1	1	1		7	
	DyrbkrD2								2		1			2				5	
	DyrbkrD3													1				1	
	DyrbkrD4													1				1	
	ElazgD1															1		1	
	ElazgD2															1		1	
	ErzrmD		1							1								2	
	EskshrD						1											1	
	HakkriD1					1						1						1	
	HakkriD2								1			1						2	
	HatayD										3	1					1	5	
	IgdrD1											1						1	
	IgdrD2											1						1	
	IsprtD1					1												1	
	IsprtD2					1												1	
	IzmrD1			3														3	
	IzmrD2			1														1	
	IzmrD3			1														1	
	IzmrD4			1														1	
	KarmanD					1												1	
	KarsD1										2							2	
	KarsD2								1		1	2						4	
	KarsD3										2							2	

Species	Haplotype	Regions																	GenBank
		**1**	**2**	**3**	**4**	**5**	**6**	**7**	**8**	**9**	**10**	**11**	**12**	**13**	**14**	**15**	**16**	**Frq**	
	KarsD4										1							1	
	KarsD5										2							2	
	KarsD6										1							1	
	KaysriD1								1								2	3	
	KaysriD2																2	2	
	KaysriD3																1	1	
	KrshrD						1											1	
	MansaD1			1						1								2	
	MansaD2			1														1	
	MansaD3			1														1	
	MansaD4			1														1	
	MarasD																1	1	
	MardnD1														2			2	
	MardnD1														2			2	
	MardnD3														1			1	
	MersnD1					1												1	
	MersnD2			1														1	
	MersnD3					1												1	
	MlatyaD1														1	3		4	
	MlatyaD2															1		1	
	MlatyaD3															1		1	
	MlatyaD4															1		1	
	MuglaD1				1													1	
	MuglaD2				1													1	
	MusD1											1	1					2	
	MusD2												1					1	
	NevshrD																1	1	

Species	Haplotype	Regions																	GenBank
		1	2	3	4	5	6	7	8	9	10	11	12	13	14	15	16	Frq	
	NigdeD						1										1	2	
	SiirtD1		1	1										1				3	
	SiirtD2						2							1				3	
	SiirtD3													1				1	
	SiirtD4													1				1	
	SiirtD5													1				1	
	SivasD								1	5	3	3			2	3	2	19	
	TekrdgD	1																1	
	TuncliD1															1		1	
	TuncliD2															1		1	
	TuncliD3															1		1	
	TuncliD4															1		1	
	UrfaD1														1			1	
	UrfaD2														1			1	
	VanD2											1						1	

Tablo S6. The D-loop 13 major haplotypes and their frequencies were used in the network analysis.

Species	Haplotype	Region																Frq
		1	2	3	4	5	6	7	8	9	10	11	12	13	14	15	16	
chukar-B	AntD1 **(E)**				4		1				1			3	1			10
	VanD1 **(m)**											2		2	1			5
chukar-A	AntD7 **(A)**			6	1	4	3		4	2		2				1	6	30
	SivasD **(B)**						1		1	5	3	3			1	3	3	20
	CnakleD6 **(C)**	7	3				1											11
	AntD5 **(D)**				1	3			2	1		1	1			1		10
	CankrD1 **(F)**			1					1		3	1					2	8
	BurdrD2 **(G)**					3		1	3									7
	DyrbkrD1 **(H)**						1		3					1	1	1		7
	AntepD4 **(i)**													2	2	1	1	6
	AntD4 **(J)**				6													6
	DyrbkrD2 **(K)**									2		1		2				5
	CnakleD5 **(L)**	2									1			2				5
