# Supplementary material for: Geographic Genetic Structure of Alectoris chukar in Türkiye: Post-LGM-Induced Hybridization and Human-Mediated Contaminations
Source: Biology (Basel). 2023 Mar 3;12(3):401. doi: 10.3390/biology12030401 (PMC10045126; doi:10.3390/biology12030401)
Supplement: Supplementary file 1 [file biology-12-00401-s001.zip › 3 - Supplementary Material S3 - Phylogeny.pdf]

Geographic genetic structure of *A. chukar* in Türkiye: Post-LGM induced hybridization and human-mediated contaminations

Sarp KAYA, Bekir KABASAKAL, Ali ERDOĞAN

Supplementary information S3: Phylogeny

Cyt-b phylogeny

The Cyt-b trees were built with 90 haplotypes obtained from this study and the sequences downloaded from NCBI (33 haplotypes out of 365 sequences), supported the monophyly of the genus (Figure S1). In the Cty-b trees, *A. melanocephala* and *A. barbara* occupied the basal branches of the tree as ancestral lineages. The branching pattern of the rest of the tree was (*A. rufa* + [*A. greaca* + ((*A. magna* + *A philbyi*) + *A. chukar*)]. The support of the *A. magna* + *A. philbyi* and (*A. magna* + *A philbyi*) + *A. chukar* nodes in the tree were found weak but the nod of the *A. greca* + [(*A. magna* + *A. philbyi*) + *A. chukar*] had high bootstrap and posterior probability values. Similarly, the monophyly of *A. chular* haplotypes received high support from the analyses but the monophyly of Clade-A and Clade-B was partially supported by phylogenetic analyses. Although the Cyt-b tree indicated that there was no contamination originating from congenerics in the *A. chukar* population in Türkiye, China Clade- B contaminations were clearly seen in six haplotypes on the tree.

The phylogenetic analyses were performed with a total of 63 haplotypes, 61 of which were from this study and 2 were outgroups obtained from NCBI. After alignment and trimming, the final length of sequences for the Cyt-b matrix was 1021 bp. Of the 1021 bp, 961 sites were constant, 60 were variable, and 17 were parsimony informative. The phylogenetic trees that were generated with 63 Cyt-b haplotypes from the *A. chukar* populations did not indicate any geographic-genetic structure within Türkiye (Figure S2). The monophyly of *A. chukar* has taken high support from all analyses but except for the Bayesian analysis, neither ML nor MP analysis supported clade- A and B as separate monophyletic lineages. The China clade-B contaminations in Türkiye were observed in the six haplotypes, and the total number of contaminated individuals was 22. This represents 8% of the total samples. The observed contamination ratio of the East (11) and West (10) parts of Türkiye was almost the same. The highest clade-B contaminations were observed in population 4 (Western Mediterranean part) with 8 individuals and the second highest value with 5 individuals in population 14 (Eastern Mediterranean part) (Figure 1).

The PCoA analysis was performed with the pairwise-Fst values obtained from 10 Cyt-b haplotypes (representing 194 individuals) on the base of the six geographic (Figure SI5). Results of the analysis showed that PC1 explains 48% of the genetic pattern of the six geographic regions, and PC2 explains only 16%. Among the six

geographic regions, Thrace was the genetically most distant area in the PCoA analysis while East, South-east, and Central Anatolia were the closest regions to each other. Mediterranean and Euxinic regions were the third geographic group genetically close to each other.

D-loop phylogeny

The D-loop phylogenetic trees that were generated by using 142 haplotypes from 305 sequences from the NCBI (34 haplotypes out of 262 sequences) and *A. chukar* sequences from this study, showed weak support for the monophyly of the genus *Alectoris* (Figure S3). In the tree, *A. melanocephala* and *A. barbara* occupied the basal branches as ancestral taxa of the genus and the branching hierarchy of the rest of the tree was (*A. rufa* + (*A. greaca* + ((*A. magna* + *A. philbyi*) + *A. chukar*))). The monophyly of the *A. chukar* haplotypes had taken good support from BI and MP analyses but not ML. The tree did not show any congenital contaminations in *A. chukar* populations, whereas China Clade-B contaminations were also confirmed. Within the species *A. chukar,* the monophyly of the Clade-A was not supported by the tree, whereas Clade-B monophyly had partial support by D-loop phylogeny. Two haplotypes AM850786 and AM850737 belong to Clade-B but they branched within the Clade-A haplotypes in D-loop phylogenetic tree.

The phylogenetic analyses were performed with a total of 171 haplotypes, 169 of which were from this study and 2 were outgroups obtained from NCBI. After alignment and trimming, the final length of sequences in the D-loop matrix with indels was 1168 bp. Of the 1168 bp, 1009 sites were constant, 116 sites were variable, 43 were gaps and 57 were parsimony informative. Without indels, the matrix included 1125 bp sites and 116 of which were variable. The D-loop phylogenetic analyses of the *A. chukar* from Anatolia did not show any geographic genetic structure within Türkiye. The tree indicated there was China Clade-B contamination in Anatolian (Figure SI 4). The D-loop tree supported the monopoly of the Clade-B haplotypes but not the clade-A haplotypes. The frequency of seven haplotypes which are represented the Clade-B contaminations in Türkiye is 17. The Clade-B contamination frequency in Eastern Türkiye is 10, while in Western Türkiye is 7, which indicates that both parts of Türkiye have almost the same contamination ratio. The D-loop haplotype network was produced using 115 haplotypes indicating a star-like phylogeny in *A. chukar* populations (Figure S5). All novel haplotypes were differentiated from the 10 ancestral haplotypes with only a few mutations, and they were spread around the ancestral haplotypes in a circular manner. This structure of the haplotype network indicates at least two sudden expansion events that happened in chukar populations in the past. The result also reveals the presence of a large number of micro-refugia in Türkiye, particularly in the Eastern part and Mediterranean. The ancestral haplotypes A and B showed regional structure. While the majority of the A haplotype sequences were coming from the Mediterranean, B haplotype was from Eastern Türkiye. The haplotype A was the most common haplotype in Türkiye. It was observed in all the regions except for Thrace and Southeast Anatolia. The second most common haplotype B, similar to haplotype A,

was not observed in Thrace and Southeast Anatolia. Haplotypes A, D, C, G, and J were widely distributed in Western Anatolia, and haplotype C was the ancestral haplotype located mostly in the Thrace region. Since B, J, L, and M haplotypes were predominantly distributed in Eastern Anatolia, it was also possible that they were sourced from this area. The analysis indicated that the Thrace population had its own unique haplotypes, and shared haplotypes mostly found in the Mediterranean part of Türkiye. The haplotype network analysis revealed the presence of China Clade-B contamination in Türkiye. The contaminants were concentrated in Mediterranean, East and Southeast Türkiye (E and m haplotypes), but there was not any Clade-B contamination in the Thrace and Euxinic part of Anatolia (Figure S5). In total, of the 17 individuals (6%) in which clade-B contaminations were detected, 7 of 17 individuals were from the West and 9 were from the East. Some haplotypes (such as L) appeared to be shared with both Thrace and Eastern and South-Eastern Anatolia. It was possible that this situation was caused by incomplete lineage sorting or human-mediated introduction. Unlike the Cyt-b gene, the D-loop region showed the presence of many ancestral and derived haplotypes originated from the Thrace region.

For the D-loop region, phylogenetic analyses were performed with a total of 307 sequences, 273 of which were from this study and 34 haploytpes (out of 262 NCBI sequences) were obtained from Genbank. After alignment and trimming, the final length of sequences in the D-loop group I matrix with indels was 1181 bp. Of the 1181 bp, 817 sites were constant, 350 were variable, 87 were gaps and 148 were parsimony informative. Without indels, the matrix included 1094 bp sites and 277 of which were variable. Phylogenetic analyses were performed with the data set which included 142 haplotypes with the length of 1094 bp using HKY+G (0.1710) substitution model showed a similar branching pattern with Cyt-b trees (SM3; Figure S3). In the D-loop phylogenetic tree, *A. melanocephala* and *A. barabara* were located in the basal branches of the tree and, *A. philbyi* + *A. magna* and *A. chukar*, were followed by these taxa respectively. Unlike the Cyt-b tree, the D-loop phylogeny supported the monophyly of clade-B but, not the monophyly of clade-A. Due to the absence of D-loop sequence of three subspecies of A. chukar (*A. c. falki, A. c. potanini* and *A. c. pubescens*) in Genbank, the branches of these taxa could not be represented in the D-loop tree. Similar to the Cyt-b tree, D-loop phylogeny indicated the presence of China clade-B contaminations in Türkiye. China clade-B contamination was detected in 17 of 273 individuals (represented by 4 haplotypes in the tree: AntD1, AntD2, AfynD1, and VanD1). The D-loop region indicated neither con-generic contamination nor any different species of genus present in Türkiye. The phylogenetic analyses performed with 171 D-loop haplotypes from Türkiye exhibited multiple polytomies and did not indicate any geographic genetic structure (SM3, Figure S4).

For the D-loop region, the network analysis was performed with 115 haplotypes and the analysis indicated a series of sudden demographic expansion events in Anatolia. The traces of these expansions might be seen around the three ancestral haplotypes of A, B, and D which are located at the centre of the haplotype network. The presence

of many old and new haplotypes was close and separated from the ancestral haplotypes by only a few mutations indicating at least two possible expansion events occurred in Türkiye (for more detail see SI 3 Figure S5). The haplotype diversity in the network analysis indicated that East and West Türkiye were the two major diversification areas for *A. chukar*.

The D-loop PCoA analysis performed with pairwise FST values of shared haplotypes among the six regions (except for the clade-B haplotypes) indicated a geographic genetic structure in Türkiye (SI 3 Figure S5). The analysis supported the existence of four geographic groups (Thrace, Mediterranean, Southeast Anatolia, and Eastern + Euxinic + Central Anatolia) within the distribution area of *A. chukar* in Türkiye. Similar to the Cyt-b analysis, D-loop PCoA also supported the Thracian part of Türkiye as the most genetically distant region within the six geographical regions. Considering PC1, Thrace and Mediterranean regions were relatively close to each other compared to other groups, while Euxinic, East, and Central Anatolia were genetically the closest regions to each other. In contrast to the Cyt-b analysis, Southeast Anatolia was separated from the East + Central Anatolia group in D-loop PCoA. Both Mt-DNA regions supported these three regions - Thrace, and Mediterranean.

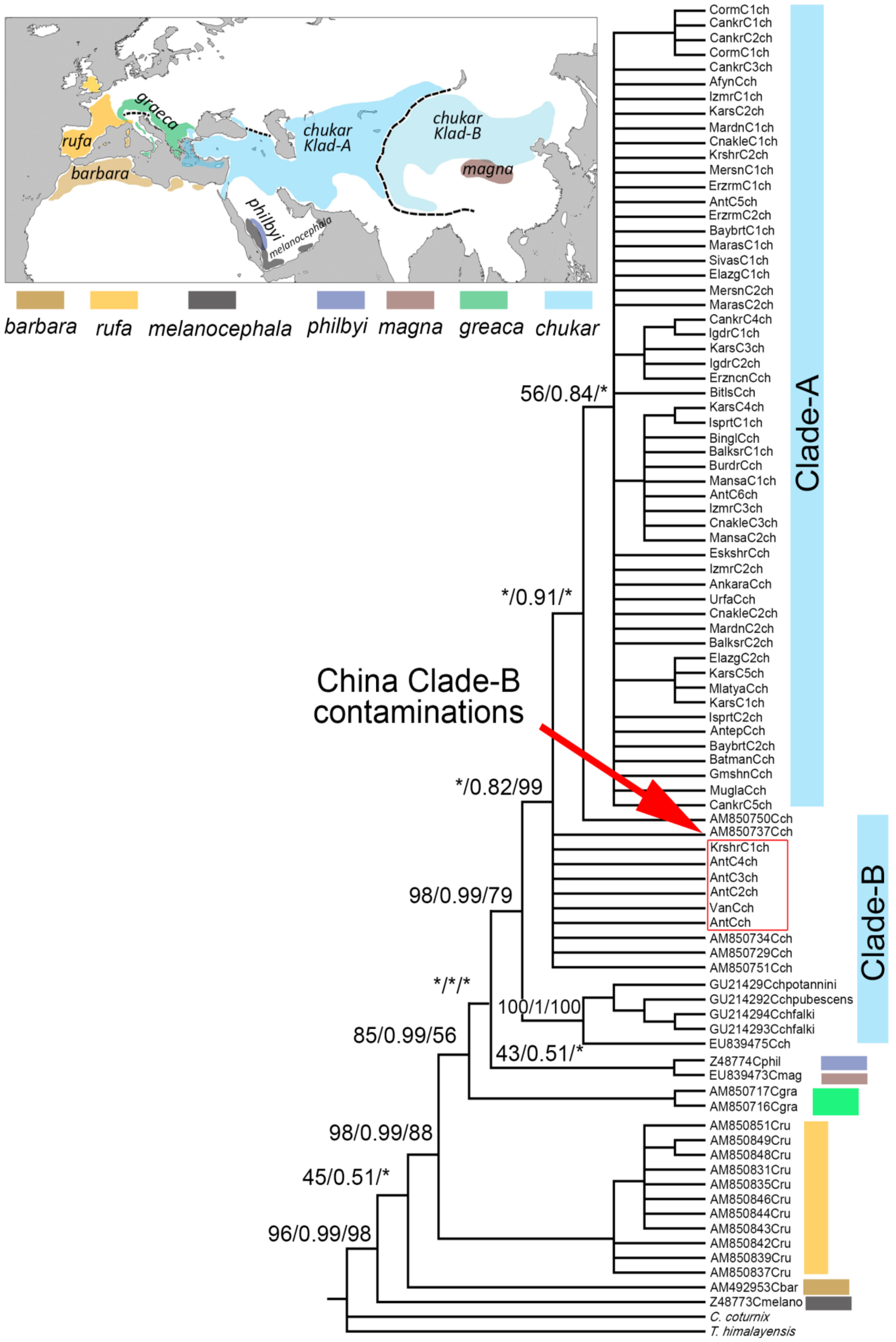

Figure S1. The Cyt-b phylogenetic tree of the genus *Alectoris* is shown on a Bayesian tree, the analyses were performed with 90 haplotypes obtained from the 325 sequences (NCBI + this project). The bootstrap values of the branches are represented ML/BI/MP analyses respectively, and the * (star) indicates the branch support values below 50%.

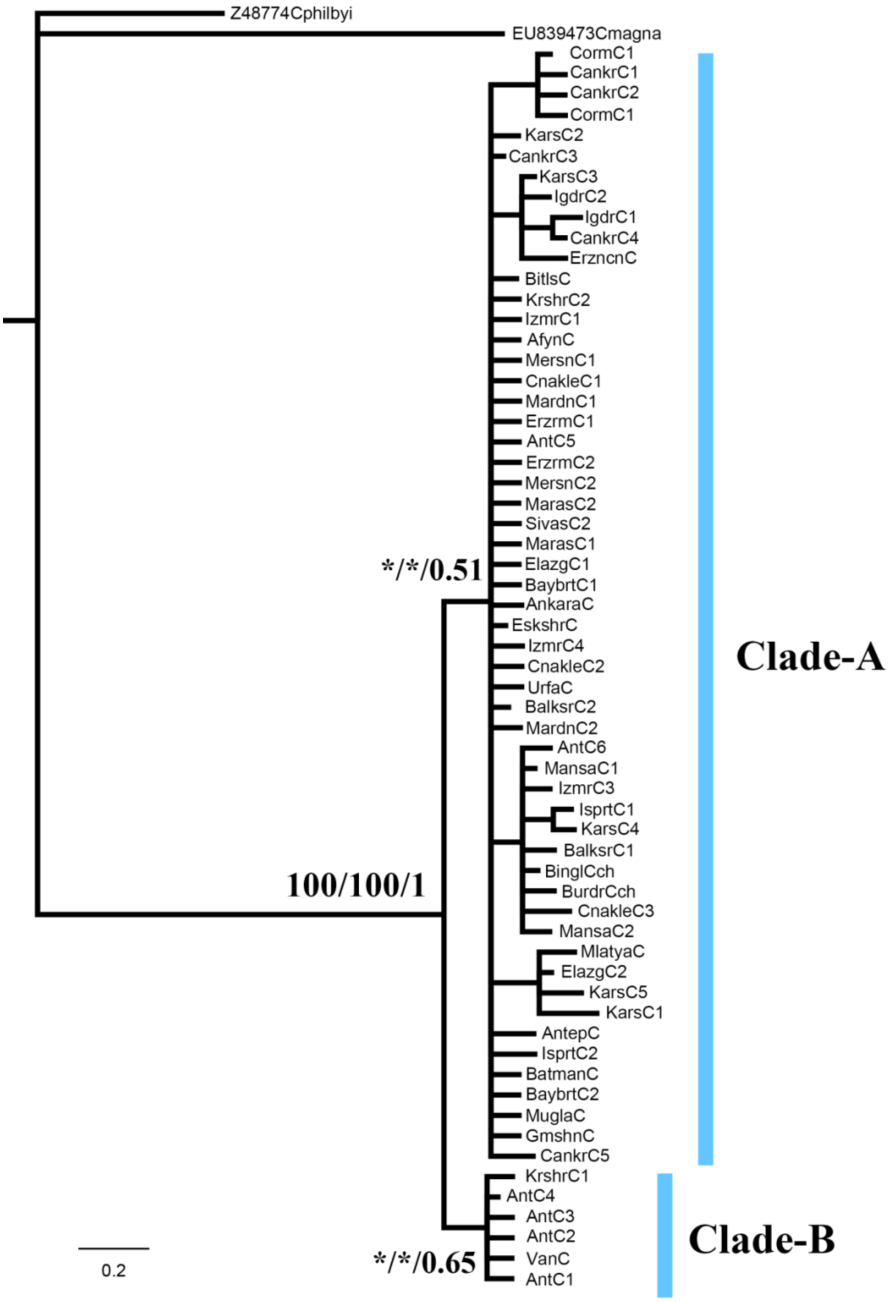

Figure S2. The Cyt-b phylogenetic tree of the *A. chukar* from Türkiye is shown on a Bayesian tree, the analyses were performed with 63 haplotypes obtained from 279 sequences. Except for the two outgroups. The rest of the sequences were obtained in this study. The values on the branches represent the ML/MP/BI branch support values, respectively, and the * (star) indicates the branch support values below 50%.

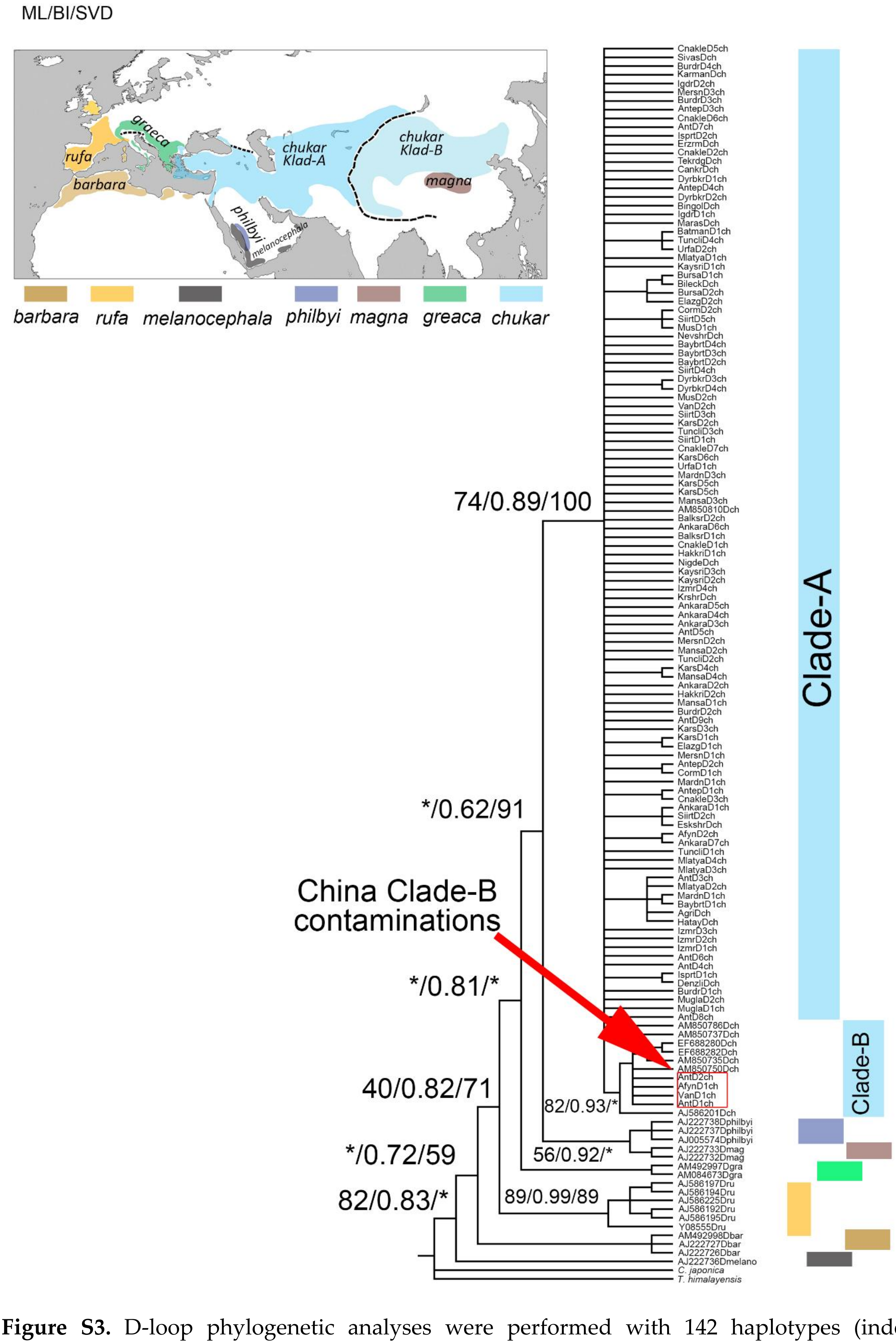

Figure S3. D-loop phylogenetic analyses were performed with 142 haplotypes (indels excluded) obtained from 305 sequences (NCBI + sequences obtained in this study). The

bootstrap values of the branches are ML/BI/MP, respectively, and the * (star) indicates the branch support values below 50%

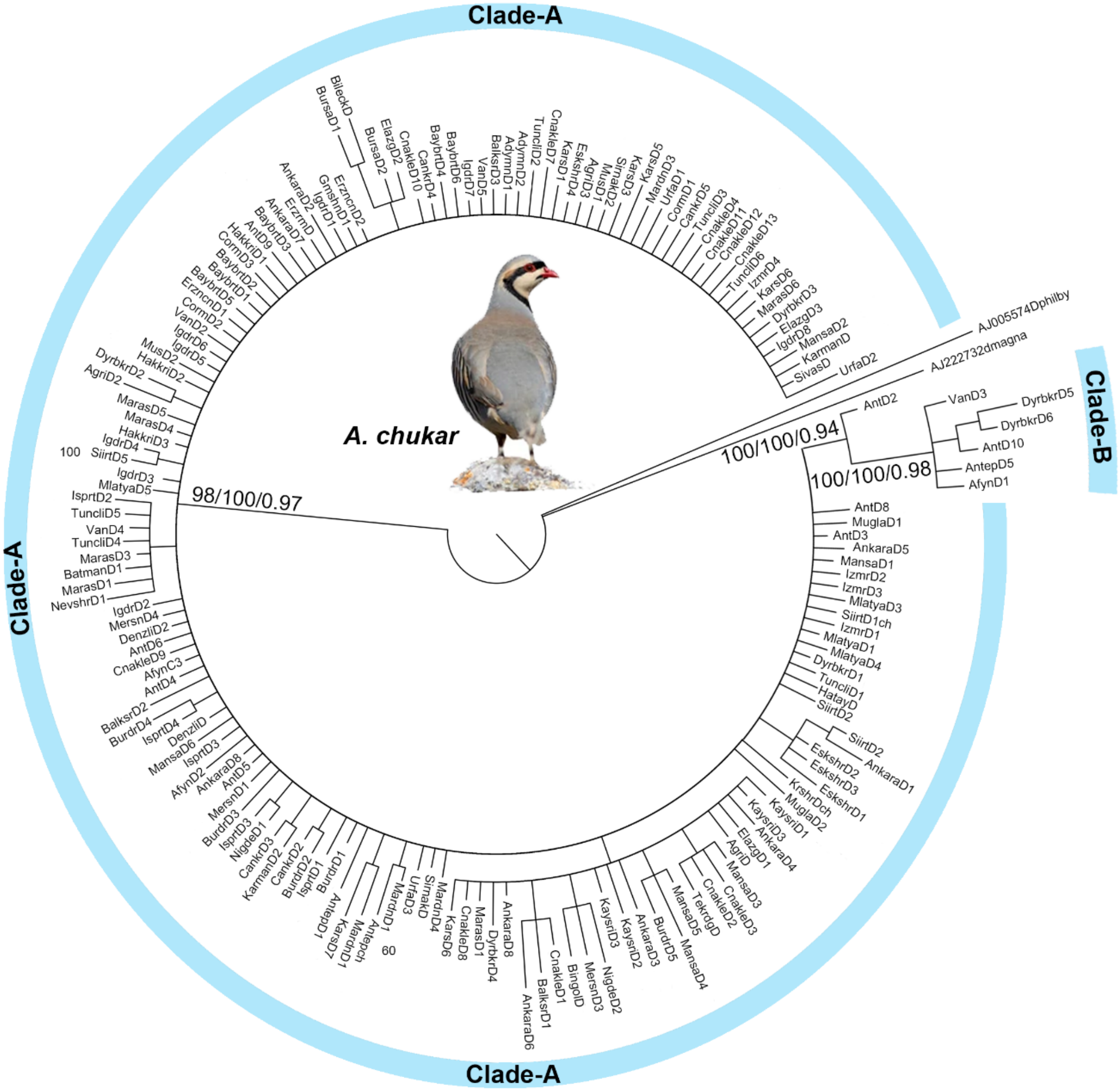

171bp -indel 1125bp

MP/ML/BI

Figure S4. The D-loop phylogenetic tree of the *A. chukar* from Türkiye is shown on a Bayesian tree, the analyses were performed with the 171 haplotypes obtained from the 275 sequences. Except the two outgroups, the rest of the sequences were obtained in this study. The values on the branches represent the ML/MP/BI branch support values, respectively, and the * (star) indicates the branch support values below 50%.

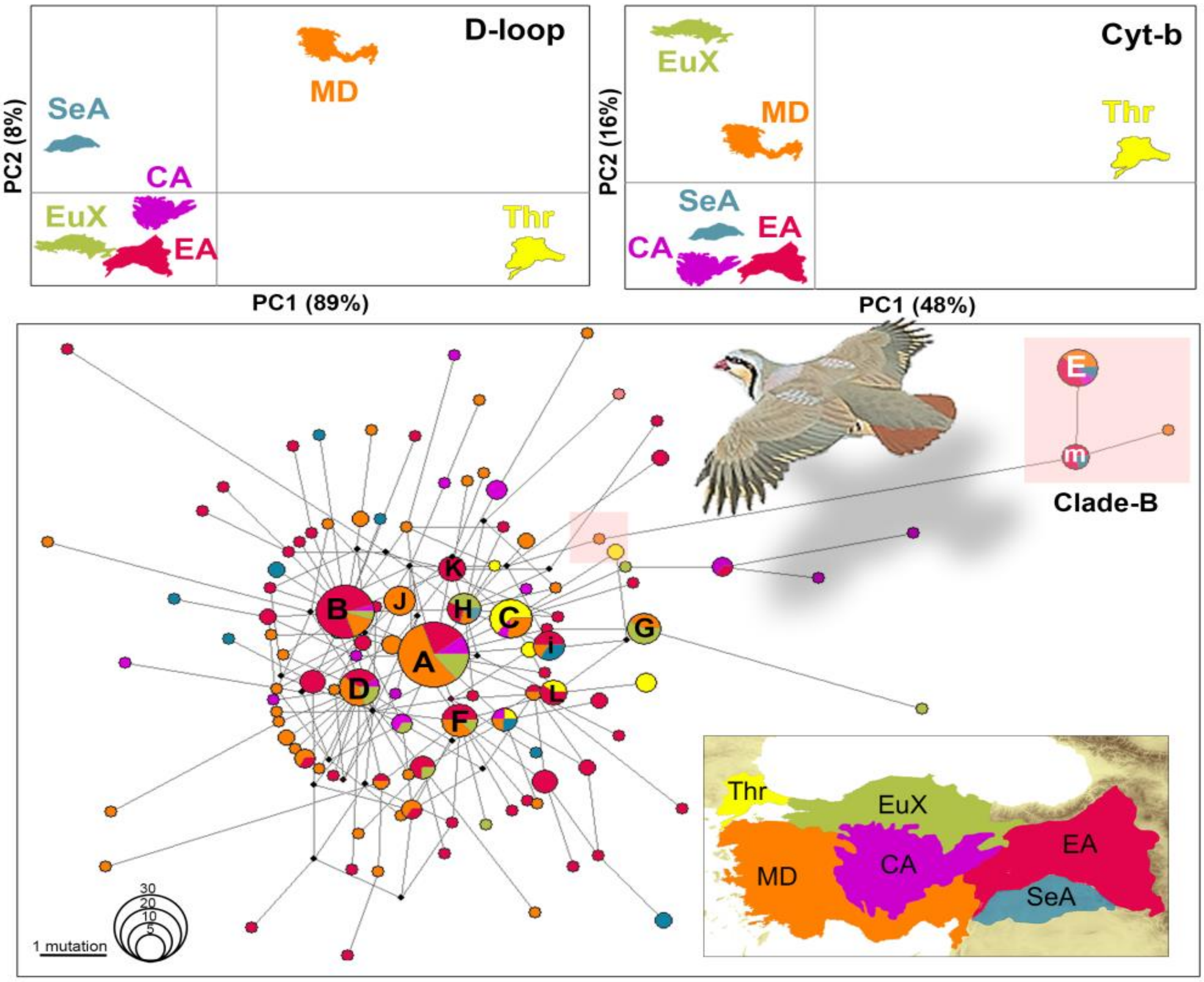

Figure S5. The D-loop Network analysis was drawn using 115 haplotypes that included 72 variable positions. PCoA analyses for D-loop and Cyt-b datasets were conducted only with shared haplotypes among the six regions.
