# Supplementary material for: Geographic Genetic Structure of Alectoris chukar in Türkiye: Post-LGM-Induced Hybridization and Human-Mediated Contaminations
Source: Biology (Basel). 2023 Mar 3;12(3):401. doi: 10.3390/biology12030401 (PMC10045126; doi:10.3390/biology12030401)
Supplement: Supplementary file 1 [file biology-12-00401-s001.zip › 4 - Supplementary Material S4 - Population genetics.pdf]

Geographic genetic structure of *A. chukar* in Türkiye: Post-LGM induced hybridization and human-mediated contaminations

Sarp KAYA, Bekir KABASAKAL, Ali ERDOĞAN

Supplementary information S4: Population genetics

Genetic diversity. Historical demography and migration rates

Table S7. The Genetic diversity indices for Cyt-b region, calculated with 277 sequences of 1024 bp length represented by 61 haplotypes from 16 regions of the *A. chukar* (Pop: population, Loc: the number of localities. N: number of samples. NoH: the number of haplotypes. UqH: the number of population-specific unshared/unique haplotypes. B: the number of China clade-B haplotypes. s: segregation sites. h: haplotype diversity. p: nucleotide diversity. d: mean number of pairwise differences of haplotypes within the population. *<0.05 **<0.0 ***<0.001)

Pop	Loc	N	No H	UqH	B	s	h±sd	π±sd	d±sd
1	2	16	3	1	0	2	0.4333+/-0.1382	0.000457+/-0.000472	0.466667+/-0.429785
2	4	9	7	4	0	7	0.9640+/-0.0770	0.002340+/-0.001589	2.388889+/-1.430426
3*	8	20	11	5	2	11	0.8632+/-0.0626	0.002160+/-0.001390	2.205263+/-1.271131
3	7	18	9	5		8	0.8366+/-0.0751	0.001671+/-0.001144	1.705882+/-1.044614
4*	6	22	11	5	8	12	0.8615+/-0.0551	0.003053+/-0.001837	3.116883+/-1.680720
4	4	14	7	3		6	0.7582+/-0.1158	0.001302+/-0.000965	1.329670+/-0.877935
5	8	22	9	4	0	8	0.8355+/-0.0551	0.001429+/-0.001005	1.458874+/-0.920024
6*	4	20	7	2	1	8	0.7684+/-0.0689	0.001815+/-0.001212	1.852632+/-1.108082
6	4	19	6	1		8	0.7427+/-0.0719	0.001604+/-0.001105	1.637427+/-1.009894
7	1	4	2	0	0	2	na	na	na
8	5	20	10	5	0	10	0.8263+/-0.0734	0.002088+/-0.001353	2.131579+/-1.237189
9	8	20	10	5	0	10	0.7650+/-0.1080	0.001299+/-0.000941	1.326316+/-0.860577
10*	1	20	10	3	1	17	0.8895+/-0.0508	0.003016+/-0.001827	3.078947+/-1.670143
10	1	19	9	3		14	0.8772+/-0.0548	0.002784+/-0.001714	2.842105+/-1.566046
11*	8	20	11	2	2	14	0.9000+/-0.0518	0.002778+/-0.001707	2.836842+/-1.560076
11	7	18	9	1		11	0.8758+/-0.0610	0.002189+/-0.001414	2.235294+/-1.291110
12	2	3	2	0	0	1	na	na	na
13*	3	20	9	1	5	11	0.8421+/-0.0607	0.002598+/-0.001615	2.652632+/-1.476107

Pop	Loc	N	No H	UqH	B	s	h±sd	π±sd	d±sd
13	2	15	7	1		7	0.7714+/-0.1001	0.001306+/-0.000962	1.333333+/-0.876172
14*	4	21	11	4	3	14	0.8762+/-0.0581	0.002500+/-0.001561	2.552381+/-1.427380
14	4	18	10	4		11	0.8497+/-0.0775	0.001876+/-0.001251	1.915033+/-1.142477
15	5	20	10	2	0	8	0.8000+/-0.0886	0.001608+/-0.001105	1.642105+/-1.009832
16	6	20	7	1	0	8	0.5842+/-0.1270	0.001046+/-0.000805	1.068421+/-0.736169

* Descriptive statistic indices were calculated with Clade-B haplotypes

The Genetic diversity parameters and descriptive statistics were calculated using the D-loop+indel dataset that included 273 sequences of 1216 bp length and excluding the data from populations 7 and 12. The genetic diversity result is consistent with the Cyt-b region and the diversity indices for all populations is quite high (SM4; Table S8). When we exclude clade-B haplotype, populations 5, 8, 11, and 15 had the highest values for haplotype diversity indices, while populations 13, 14 and 16 had the highest values for nucleotide diversity. The populations 6, 14, and 16 showed the highest value of the mean number of pairwise differences between the haplotypes within a population, which means these areas might be the possible mixing areas for *A. chukar* individuals in Türkiye. The D-loop data also support that population 4 is the most contaminated population in Anatolia with the 5 haplotypes from China clade-B. Population 1 exhibited the lowest genetic diversity values in all parameters, which was similar to the Cyt-b. However, population 16 shows high values in the big part of the parameters, the differences between the two genetic markers could be due to the accumulation of different neutral mutation rates between D-loop and Cyt-b. Nevertheless, both mitochondrial markers harmoniously indicate that population 1 (Thrace population) has lived through a strong bottleneck.

Table S8. The Genetic diversity indices for D-loop region, calculated with 273 sequences of 1216 bp length includes indel sites represented by 115 haplotypes from 16 regions of the *A. chukar* (Pop: population, Loc: the number of localities. N: number of samples. NoH +indel: the number of haplotypes include indel sites. NoH –indel: the number of haplotypes without indel sites. UqH: the number of population-specific unshared/unique haplotypes. B: the number of China clade-B haplotypes. s: segregation sites. NoID: the number of indel sites. h: haplotype diversity. p: nucleotide diversity. d: mean number of pairwise differences of haplotypes within the population. *<0.05 **<0.0 ***<0.001) (115 haplotypes were obtained from 273 sequences the D-loop region. but the presence of 151 haplotypes in the table is due to the independent reintroduction of shared haplotypes into each population).

Pop	Loc	N	NoH +indel	NoH -indel	UqH	B	s	NoI D	h±sd	π±sd	d±sd
1	3	18	12	6	12	0	18	9	0.8954+/-0.0653	0.00321+/-0.0019	3.8497+/-2.0288
2	4	9	8	7	9	0	22	7	0.9722+/-0.0640	0.00609+/-0.0036	7.3056+/-3.7773

Pop	Loc	N	NoH +indel	NoH -indel	UqH	B	s	NoID	h±sd	π±sd	d±sd
3*	8	20	16	13	16	1	37	12	0.9737+/-0.0250	0.00568+/-0.0031	6.8263+/-3.3552
3	8	19	15	13	15		32	12	0.9708+/-0.0273	0.00509+/-0.0028	6.117+/- 3.0447
4*	6	20	15	11	15	5	27	9	0.9579+/-0.0328	0.00551+/-0.003	6.6105+/-3.2586
4	5	15	11	10	10		14	3	0.9333+/-0.0538	0.00278+/-0.0017	3.3333+/-1.8119
5	12	20	20	15	20	0	40	23	1.0000+/-0.0158	0.00682+/-0.0037	8.2263+/-3.9812
6*	7	20	19	17	18	1	43	13	0.9947+/-0.0178	0.00727+/-0.0039	8.7158+/-4.1999
6	7	19	19	17	11		37	11	0.9942+/-0.0193	0.0067+/-0.0036	8.0526+/- 3.9128
7	3	4	4	4	3	0	9	0	na	na	na
8	5	20	16	10	14	0	25	8	1.0000+/-0.0158	0.004718+/-0.0026	5.6474+/-2.8271
9	8	20	14	12	14	0	17	4	0.9474+/-0.0344	0.00264+/-0.0016	3.1526+/-1.7035
10*	1	20	13	11	12	1	37	16	0.9579+/-0.0255	0.00509+/-0.0028	6.1579+/-3.0559
10	1	19	12	9	11		33	16	0.9532+/-0.0276	0.00458+/-0.0026	5.5322+/-2.782
11*	5	20	20	15	19	2	29	6	1.0000+/-0.0158	0.00529+/-0.0029	6.3368+/-3.1361
11	5	18	18	13	17		23	6	1.0000+/-0.0185	0.0044+/-0.0025	5.2745+/-2.673
12	2	4	4	4	3	0	15	2	na	na	na
13*	5	20	19	14	19	5	41	18	0.9947+/-0.0178	0.00847+/-0.0045	10.1684+/- 4.8485
13	5	15	14	12	14		27	12	0.9905+/-0.0281	0.00665+/-0.0037	7.9714+/-3.926
14*	4	20	16	16	16	2	40	12	0.9789+/-0.0214	0.0076+/-0.0041	9.1105+/-4.3762
14	4	18	14	13	14		35	12	0.9739+/-0.0253	0.00681+/-0.0037	8.1634+/-3.9728
15	5	20	20	16	20	0	33	13	1.0000+/-0.0158	0.0062+/-0.0034	7.4368+/-3.6283
16	5	18	16	9	16	0	29	9	0.9869+/-0.0229	0.00732+/-0.004	8.7778+/-4.2487

* Descriptive statistic indices were calculated with Clade-B haplotypes

Tablo S9. D-loop based historical demographic estimates of 14 populations.

Pop	N	Hs	Neutrality tests		*g*	Mismatch distribution				
		+indel	Tajima's D	Fu's Fs		θ_0	θ_1	τ	SSD	HRI
1	18	12	-0.41955	-4.57256**	952.8662	1.932	9.05	2.93	0.0062019	0.0203341
2	9	8	-0.8686	-1.74255	1191.864	0.007	63.043	8.063	0.0333873	0.091821
3	20	16	-1.45334	-6.14776**	1019.089	2.535	90.152	4.645	0.0081731	0.0255125
4*	20	15	0.04686	-4.84206*	552.6925	7.2	4074.99	1.25	0.0152426	0.0221053
4	15	11	-0.83782	-5.18575**	605.3439	0.55	6934.956	2.875	0.0126471	0.058322
5	20	20	-0.70464	-14.17704***	1817.468	3.6	7254.95	6	0.004657	0.0107479
6	20	19	-1.5224	-10.50857***	2530.101	3.217	52.148	6.566	0.0058646	0.0123823

Pop	N	Hs	Neutrality tests		g	Mismatch distribution				
		+indel	Tajima's D	Fu's Fs		Θ_0	Θ_1	τ	SSD	HRI
8	20	16	-0.87916	-18.04223***	2797.519	1.9	3414.98	4	0.0053694	0.0135734
9	20	14	-1.19019	-8.12563***	3435.406	0.014	70.077	3.41	0.0018654	0.0252078
10*	20	13	-1.04396	-2.79673	885.8764	4.141	165.94	2.178	0.0102557	0.0307479
10	19	12	-1.00089	-2.5382	1219.8	2.849	87.656	2.469	0.0110584	0.0359085
11*	20	20	-0.93047	-16.79670***	1415.849	2.8	3414.98	4	0.0013531	0.0094183
11	18	18	-0.93316	-15.79589***	2853.973	1.9	3414.978	4	0.0048323	0.0188816
13*	20	19	-0.1629	-9.27927**	945.3481	5.068	705.01	6.293	0.0060658	0.0112188
13	15	14	-0.4489	-6.12320**	1780.337	5.5	3414.978	6	0.01872455*	0.0164172
14*	20	16	-0.9596	-4.43682*	1031.127	3.215	37.988	7.303	0.0056651	0.0116344
14	18	14	-1.04363	-3.51785	1284.897	2.132	29.321	7.203	0.0082232	0.018369
15	20	20	-1.41722	-15.15624***	3812.448	3.6	7254.95	5	0.0108849	0.0233795
16	18	16	-0.69219	-6.23319**	2671.819	0.005	46.264	12.152	0.0213232	0.0272545

* Descriptive statistic indices were calculated with Clade-B haplotypes

Explanations: Shown genetic diversity indices in populations from up to down are; Watterson Theta (θ). Under a model of sudden population expansion. Tajima's D. Fu's FS and Fu and Li's D* are expected to be significantly negative. Mismatch analysis parameters: θ0 is the substitution rate before the expansion and θ1 is the substitution rate after the expansion. expansion parameter τ. (with lower and upper bounds at $\alpha = 0.05$). SSD tests the validity of a stepwise expansion model based on the sum of squares deviations between the observed and expected mismatch. with probability values (P). Non-significant mismatch values suggest population expansion. Harpendin's Raggedness index is calculated similarly. and with probability values (P). Non-significant mismatch values suggest population expansion.

The GMRF-Skyride plot analyses showed a gradual demographic expansion in all *A. chukar* populations except populations 1, 2, and 6. The expansion events accelerated particularly at the end of the LGM (~22 kyBP) (SM4; Figure S7). TMRCA estimation for each population dated back to the late Pleistocene, with mean values between 0.03 – 0.125 myrBP. The TMRCA of population 1 with 35 kyBP was the most recent time estimation among the populations. This result indicates that population 1 lost the majority of its genetic diversity, and it passed through a recent time bottleneck. The LAMARC migration analysis results revealed that the highest historical gene flow values among the six regions were observed in Central Anatolia and nearby regions, especially between CA-MED and CA-EA (SM4; Table S10). Only the value of a migration rate over 0.8 was detected in CA and EA (812) regions. The later highest value was observed between CA and MED (768) regions (SM4; Table S10). The migration analysis shows there is a migration bias in Türkiye, high-level migration from MED to CA and EA to CA indicate a high-level gene flow at the centre of Anatolia occurred from both sites. The second highest rate of gene flow was detected among population 1 with regions MED (703) and EuX (559), respectively.

The genetic diversity and historical demographic analyses that were performed with the D-loop region supported high genetic diversity and demographic expansions in *A. chukar* populations in Türkiye (SM4; Table S8-S9). Fu's Fs tests indicated strong demographic expansions in all *A. chukar* populations, even in populations 1 and 6, in contrast to the Cyt-b gene. The demographic expansion is quite severe in populations 5, 6, 8, 9, 11 and 15, respectively. The g parameter and mismatch distribution analyses also

supported a similar situation within Türkiye. The g parameter results indicated that there is a demographic expansion in all populations and the fastest growth was observed in populations 8, 9, 11, 15, and 16, respectively (SM4; Table S9).

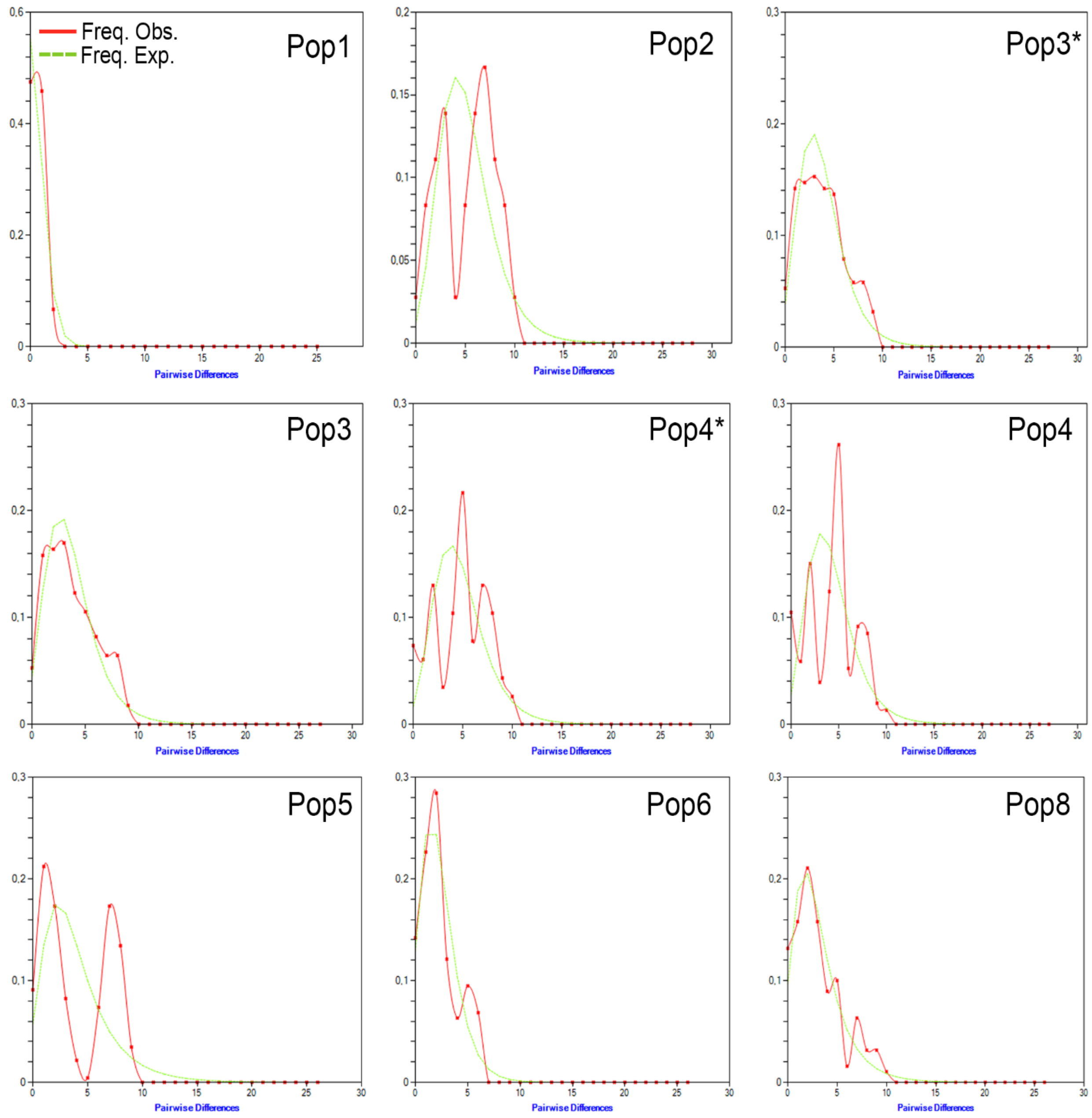

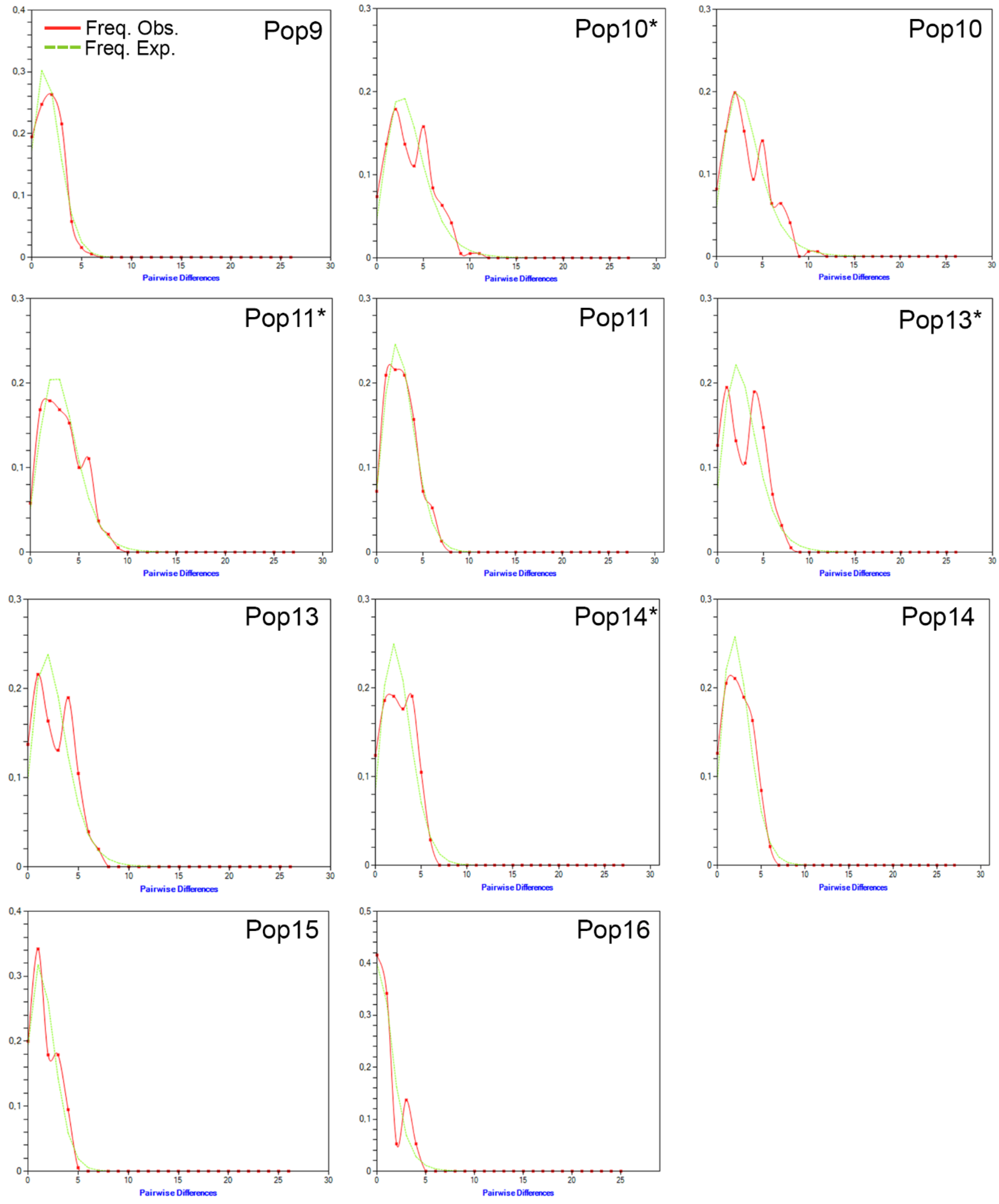

Figure S6. Mismatch distributions depicting the demographic history for each population of *A. chukar* using Cyt-b gene sequence in Türkiye. The red lines show the observed frequency distribution while the dashed lines show the distribution expected under the sudden-expansion model. The horizontal axis is the genetic distance between the sequences, and the vertical axis is the logarithmic (LogNef) representation of the effective population size. * Descriptive statistic indices were calculated with Clade-B haplotypes

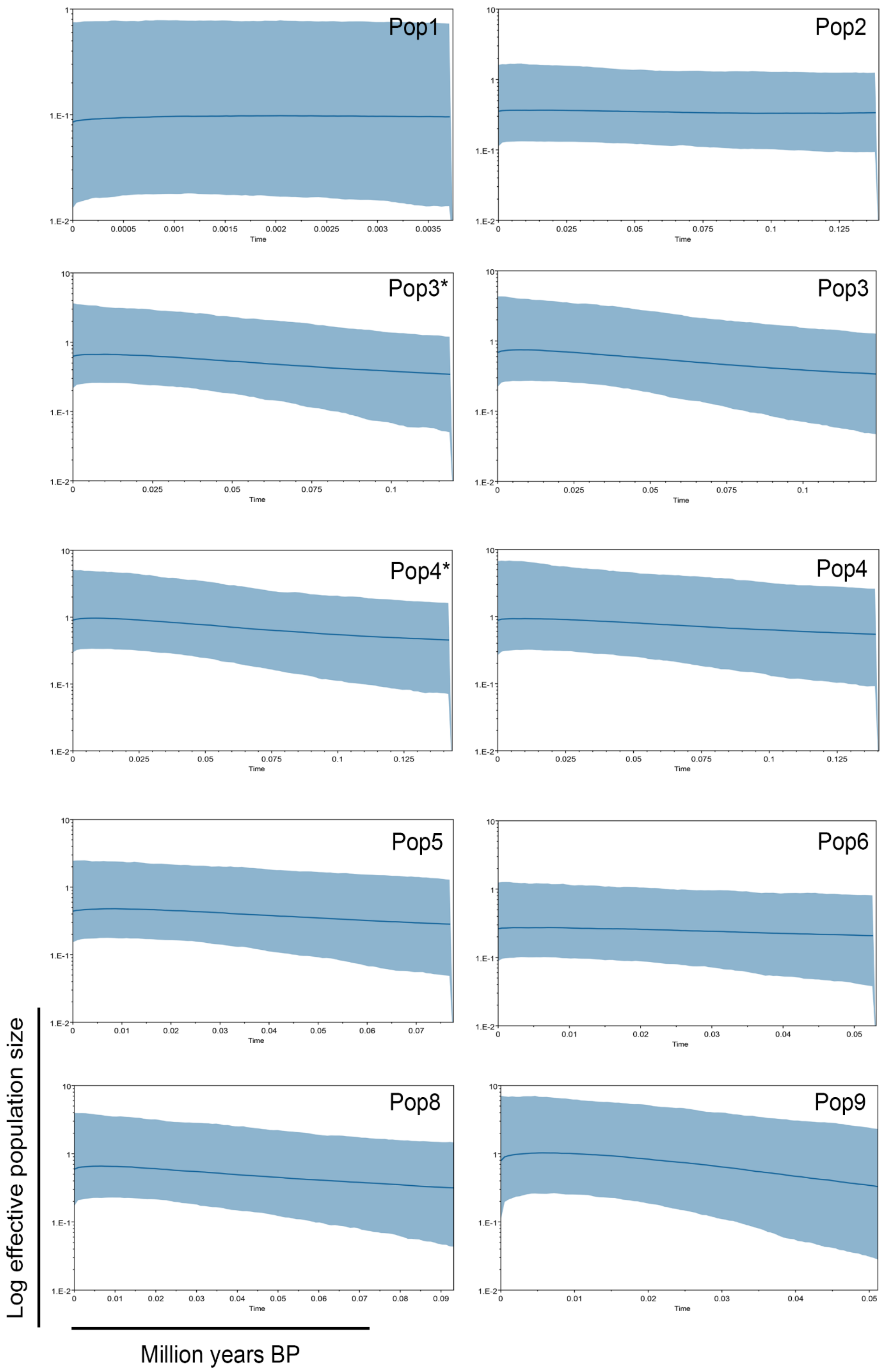

Pop1
Pop2
Pop3*
Pop3
Pop4*
Pop4
Pop5
Pop6
Pop8
Pop9
Time
Log effective population size
Million years BP

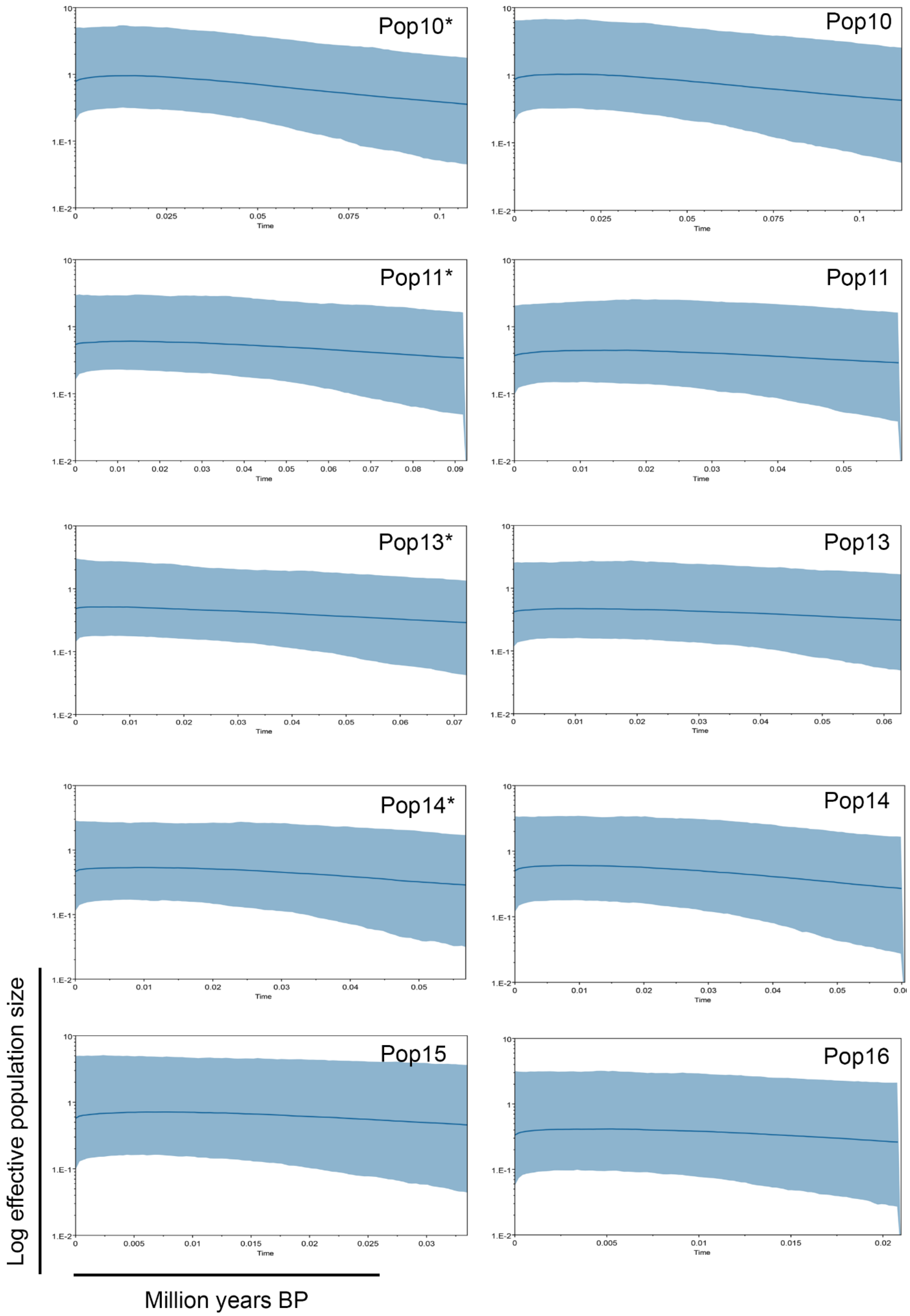

Figure S7. Historical demographic history of the 14 populations with and without clade B haplotypes as estimated with GMRF Bayesian skyride plots. Thick line shows the median predicted population size (Ne x τ; τ = generation length in units of time) on logarithmic scale; the shaded area represents the 95% highest posterior density (HPD), and lines indicate median estimates of the time to the most recent common ancestor. The horizontal axis represents the time (Million years) and the vertical axis represents the logarithmic (LogNef) equivalent of the effective population size.

Table S10. Bayesian estimates of historical asymmetrical migration using between 6 regions of *A. chukar* populations, based on Cyt-b regions by using the program LAMARC-migrate module. (1: Thrace, 2: Euxinic, 3: Central Anatolia, 4: Mediterranean, 5: East Anatolia, 6: Southeast Anatolia)

**Parameter**	**0.03**	**0.25**	**Mode**	**0.75**	**0.98**	**Median**	**Mean**
M1->2	619.33	662.00	665.00	665.33	692.67	587.67	559.03
M1->3	0.00	0.00	1.00	3.33	212.67	100.33	126.60
M1->4	730.00	908.67	910.33	910.67	999.33	737.67	702.87
M1->5	3.33	46.00	73.67	106.00	226.00	247.67	355.84
M1->6	416.00	424.00	425.00	426.00	428.67	482.33	495.92
M2->1	0.00	8.67	13.00	14.67	164.67	146.33	231.01
M2->3	0.00	0.00	4.33	36.67	128.67	42.33	63.41
M2->4	0.00	0.00	10.33	30.67	178.00	59.00	146.02
M2->5	0.00	2.67	7.67	34.67	169.33	92.33	142.72
M2->6	0.00	12.00	21.67	27.33	104.67	292.33	351.11
M3->1	558.00	588.67	591.67	592.00	606.67	620.33	584.85
M3->2	804.67	958.67	961.00	969.33	999.33	716.33	654.24
**M3->4**	870.67	930.67	933.67	936.67	999.33	818.33	**767.74**
**M3->5**	737.33	997.33	999.67	999.33	999.33	838.33	**812.37**
M3->6	745.33	909.33	917.00	917.33	999.33	752.33	698.94
M4->1	0.00	0.00	1.00	29.33	216.67	83.67	127.07
M4->2	0.00	0.00	55.67	56.00	234.00	181.67	264.65
**M4->3**	0.67	43.33	49.67	52.00	184.67	93.67	**115.39**
M4->5	0.00	0.00	0.33	31.33	133.33	39.67	63.82
M4->6	79.33	115.33	116.33	116.67	126.67	351.00	380.37
M5->1	0.00	0.00	9.67	38.00	215.33	75.00	126.01
M5->2	194.00	204.00	205.00	206.00	206.67	527.00	524.81
**M5->3**	0.00	0.00	3.00	26.67	124.00	40.33	**60.71**
M5->4	218.00	264.00	267.00	268.00	344.67	367.00	399.08
M5->6	570.00	586.00	587.00	587.33	589.33	691.00	661.21
M6->1	0.00	0.00	5.00	22.00	216.00	114.33	162.21
M6->2	0.00	11.33	19.67	26.00	296.00	218.33	299.96
M6->3	0.00	0.00	1.00	39.33	136.00	51.00	88.91
M6->4	0.00	0.00	2.33	39.33	206.67	69.67	118.12
M6->5	10.00	72.00	77.00	84.67	206.67	118.33	140.48
