# Supplementary material for: Geographic Genetic Structure of Alectoris chukar in Türkiye: Post-LGM-Induced Hybridization and Human-Mediated Contaminations
Source: Biology (Basel). 2023 Mar 3;12(3):401. doi: 10.3390/biology12030401 (PMC10045126; doi:10.3390/biology12030401)
Supplement: Supplementary file 1 [file biology-12-00401-s001.zip › 5 - Supplementary Material S5 - Microsatellite.pdf]

Geographic genetic structure of *A. chukar* in Türkiye: Post-LGM induced hybridization and human-mediated contaminations

Sarp KAYA. Bekir KABASAKAL. Ali ERDOĞAN

Supplementary information S5: Microsatellite

Microsatellite markers

The genotyped individuals per locus vary from 250 to 322. and the highest number of genotyped individuals was observed at locus MCW135. Among the 13 microsatellite markers. the highest allelic variations were detected at Aru1E97, Aru1E45. and Aru1.27 and the lowest allelic variation was observed at MCW280 and MCW118. The results of Fisher's exact test for HW equilibrium showed significant deviations for all populations. Significant deviations from HWE were detected in all markers except Aru1E7. The highest expected heterozygosity (HE) was found at locus Aru1E97 (0.946). and the lowest value was detected at locus MCW280 (0.06444).

Table S11. Summary of genetic diversity indices for each of 13 microsatellite loci studied in the study. (Na: mean number of alleles per locus. N: Number of genotyped partridges. H_O: observed heterozygosity and H_E: expected heterozygosity. Ar: allele richness).

**No**	**Locus**	N_A	*N*	H_O	H_E	**Ar**	**Size range (bp)**
1	MCW118	2.875±1.408	259	0.30251	0.31953	11	125-157
2	MCW0121	6.000±1.932	298	0.69770	0.69743	9	184-202
3	MCW135	4.875±1.500	322	0.61207	0.66334	8	106-120
4	MCW225	3.312±1.250	275	0.52946	0.54985	9	139-163
5	MCW276	4.562±1.365	297	0.33893	0.53172	8	191-203 (217)
6	MCW280	1.625±1.088	265	0.05088	0.06444	6	163-177
7	Aru1.23	3.125±0.619	318	0.48527	0.44440	6	177-187
8	Aru1.27	8.688±2.496	283	0.68670	0.80238	16	170-206
9	Aru1E97	16.812±5.856	283	0.91637	0.94634	41	209-401
10	MCW146	3.938±1.340	307	0.57108	0.60441	6	149-159
11	Aru1E7	6.812±1.642	296	0.74675	0.80255	13	179-211
12	Aru1E45	9.125±3.914	292	0.55258	0.66969	23	134-182
13	MCW0069	3.062±1.389	250	0.26870	0.35131	8	143-165

Table S12. Summary of genetic diversity indices in *A. chukar* populations at 13 microsatellite loci. (Pop: Population. PL: polymorphic locus. Na: mean number of alleles per locus. N: Number of genotyped partridges. H_O: observed heterozygosity and H_E: expected heterozygosity. Ar: allele richness. F_{IS}: fixation index).

Pop.	*PL*	*Na*	*N*	*Ho*	*He*	Ar	F_{IS}
1	11	4.364±2.292	17	0.39735±0.33695	0.49884±0.25958	9.727±11.455	0.3439**
2	12	3.917±2.234	10	0.57477±0.22667	0.61872±0.23754	9.417±14.582	0.0592*
3	12	5.750±4.224	22	0.46840±0.22861	0.55605±0.24827	11.000±15.650	0.1545***
4	13	5.846±4.506	24	0.50781±0.28536	0.56746±0.24880	13.154±23.021	0.1005***
5	13	6.692±5.170	24	0.46136±0.26051	0.53662±0.25960	13.385±19.662	0.1805***
6	12	6.250±5.048	23	0.54696±0.23609	0.63799±0.21074	12.000±19.207	0.1267***
7	10	3.700±1.829	4	NA	NA	NA	NA
8	13	6.154±4.879	21	0.48621±0.26284	0.55733±0.26489	11.462±17.539	0.1498**
9	13	7.077±4.252	33	0.57504±0.23463	0.68522±0.14489	11.385±14.886	0.1949***
10	12	6.833±4.988	26	0.59147±0.13204	0.66186±0.15879	12.250±15.938	0.1075**
11	13	6.923±3.796	27	0.53266±0.27036	0.61594±0.24047	13.692±14.767	0.1977***
12	11	3.636±1.748	4	NA	NA	NA	NA
13	12	7.250±5.379	29	0.59747±0.27289	0.65764±0.18885	12.083±16.110	0.1337***
14	13	7.231±4.850	28	0.57471±0.24278	0.61523±0.21965	11.692±17.221	0.0681**
15	12	7.417±5.961	29	0.53678±0.23781	0.63469±0.25659	13.333±19.523	0.1498***
16	12	7.667±5.990	26	0.65347±0.22631	0.65858±0.20580	12.500±17.160	0.0023**

$^{*}P<0.05$. $^{**}P<0.01$. $^{***}P<0.001$

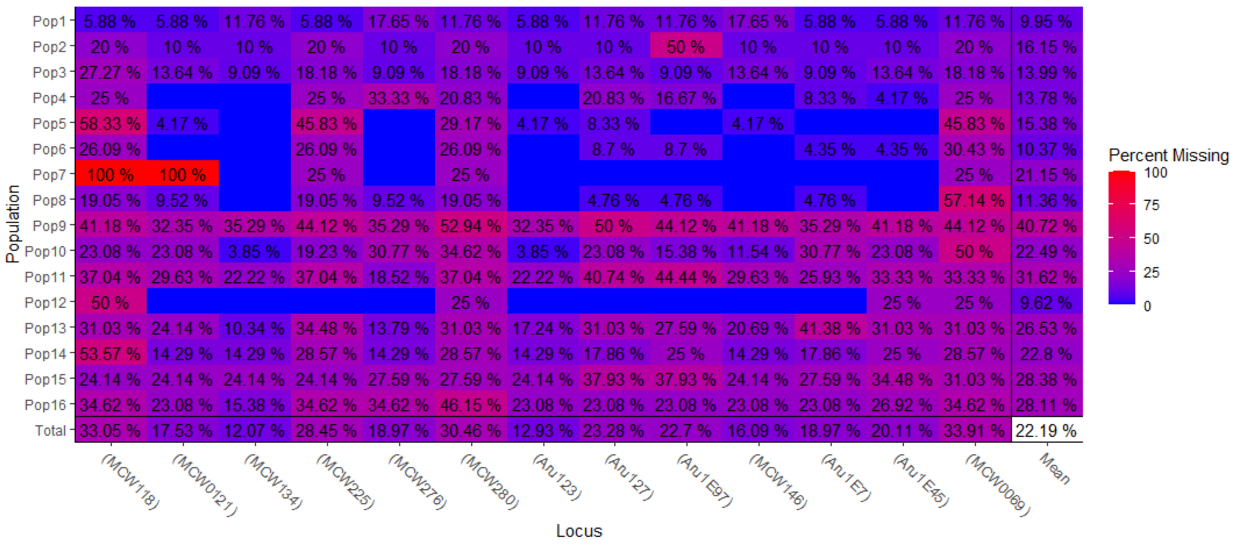

Figure S8. The missing value of loci and populations

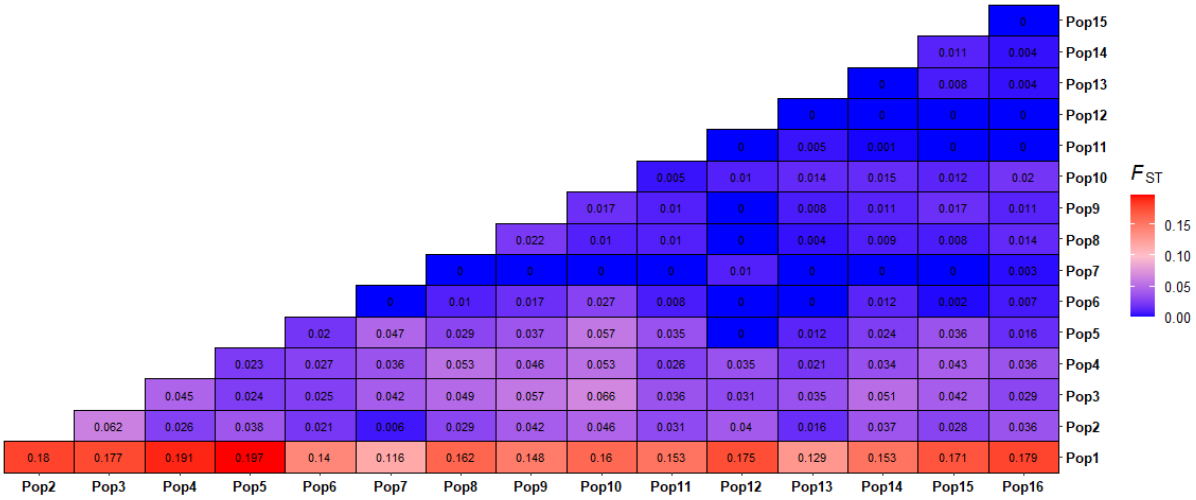

Figure S9. Pairwise F_{ST} results of 16 *A. chukar* populations

Table S13. Structure harvester analysis results and Delta K values.

K	Reps	Mean LnP(K)	Stdev LnP(K)	Ln'(K)	|Ln''(K)|	Delta K
2	10	-10744.7	8.3855	NA	NA	NA
3	10	-10604.1	24.2305	140.6	70.12	2.893878
**4**	**10**	**-10533.6**	**26.8237**	**70.48**	**116.09**	**4.327895**
5	10	-10579.2	53.6984	-45.61	38.91	0.724603
6	10	-10585.9	142.9524	-6.7	38.97	0.272608

7	10	-10631.6	84.99	-45.67	202.5	2.382634
8	10	-10879.8	308.6792	-248.17	14.76	0.047817
9	10	-11113.2	526.4206	-233.41	848.23	1.611316
10	10	-12194.8	1396.488	-1081.64	NA	NA
