# Supplementary material for: Geographic Genetic Structure of Alectoris chukar in Türkiye: Post-LGM-Induced Hybridization and Human-Mediated Contaminations
Source: Biology (Basel). 2023 Mar 3;12(3):401. doi: 10.3390/biology12030401 (PMC10045126; doi:10.3390/biology12030401)
Supplement: Supplementary file 1 [file biology-12-00401-s001.zip › 6 - Supplementary Material S6 - DIYABC.pdf]

Geographic genetic structure of *A. chukar* in Türkiye: Post-LGM induced hybridization and human-mediated contaminations

Sarp KAYA, Bekir KABASAKAL, Ali ERDOĞAN

Supplementary information S6: ABC

Table S14. The prior parameter ranges were arranged for ABC analysis. N: effective population size, t: number of generations, ra: admixture ratios. (Generation time for *A. chukar* is 3.9 year/generation)

Parameters	Minimum values	Maximum values	Conditions
N1, N2, N3, N4	10^3	10^6	
Na, N5, Nd	10^4	$5x10^6$	
t1, t4, t6	$1.5x10^3$	$288x10^3$	
t2, t7, t3, t5, t8	$12.5x10^3$	$288x10^3$	
Na, N5, Nd			> N1, N2, N3, N4
t2, t7			> t1, t4, t6
t3, t5, t8			> t1, t4, t6, t2, t7
ra	0.001	0.999	

Table S15. The parameters calculated based on hypothesis 1 (Generation time for *A. chukar* is 3.9 year/generation).

Parameter	Mean	Median	Mode	Q 0.25	Q 97.5
N1	1.89e+003	1.75e+003	1.69e+003	1.27e+003	2.85e+003
N2	1.29e+005	8.56e+004	5.40e+004	2.13e+004	5.41e+005
N3	1.12e+005	6.69e+004	3.08e+004	1.46e+004	5.43e+005
N4	4.55e+004	2.49e+004	1.38e+004	6.29e+003	2.51e+005
t1	2.07e+003	1.87e+003	1.61e+003	1.53e+003	3.80e+003
ra	3.30e-001	2.74e-001	2.28e-001	1.47e-002	9.07e-001
t2	1.25e+004	1.22e+004	1.19e+004	1.17e+004	1.26e+004
Na	1.25e+006	7.64e+005	3.27e+004	3.60e+004	4.46e+006
t3	1.29e+004	1.26e+004	1.25e+004	1.25e+004	1.43e+004
