# Supplementary material for: Geographic Genetic Structure of Alectoris chukar in Türkiye: Post-LGM-Induced Hybridization and Human-Mediated Contaminations
Source: Biology (Basel). 2023 Mar 3;12(3):401. doi: 10.3390/biology12030401 (PMC10045126; doi:10.3390/biology12030401)
Supplement: Supplementary file 1 [file biology-12-00401-s001.zip › 7 - Supplementary Material S7 - ENM.pdf]

Geographic genetic structure of *A. chukar* in Türkiye: Post-LGM induced hybridization and human-mediated contaminations

Sarp KAYA, Bekir KABASAKAL, Ali ERDOĞAN

Supplementary information S7: Ecological Niche Modelling

The Present and Last Glacial Maximum (LGM) climate data were obtained from a general circulation model (GCM) based bioclimatic layers. The original GCM data were obtained from the WorldClim website (http://www.worldclim.org/) with a spatial resolution of 30 arcseconds for the Present and 2.5 arc-minutes for the LGM. To avoid highly correlated and redundant climatic variables in our climatic dataset, which can cause over-parameterization and multicollinearity in analyses [186], we applied biolayer elimination strategies using Pearson's correlation coefficient with ENMTOOLS V.1.3 [187,188]. When two variables shared a correlation coefficient of 0.80 or higher, these were considered highly correlated, and the most meaningful variable was selected according to the present contribution in the ENM analysis. The default convergence threshold was used in MAXENT 3.3.3k [128,129], maximum iterations were increased to 1000 to allow the model time to converge, and regularization values and functions of environmental variables were selected automatically. The modelled distributions were generated with 75% of the points (training data) and cross-validated with 25% of the remaining localities (test data), averaged over 15 runs. Model performance was evaluated using the area under the curve (AUC) and the threshold-dependent binomial omission tests calculated by MAXENT. The model predictions were visualized using ArcGIS v.10.7.1.

Table S16. The area under the curve (AUC) and the contribution of biolayers for each of ENMs

LGM (CCSM) (AUCmean=99.6)		LGM (MIROCH) (AUCmean=99.6)		Present (AUCmean=99.1)	
Biolayers	% contribution	Biolayers	% contribution	Biolayers	% contribution
18	68.5	9	38.9	9	25.8
7	17.6	7	13.8	19	24.8
1	10.2	13	13.3	4	14.1
9	2.8	1	11.8	8	13.7
8	0.9	8	11.5	15	11.2
3	0	17	8	17	6.8
10	0	15	2.7	3	1.9
19	0	2	0	11	1.1
		10	0	10	0.6

References

Buermann, W.; Saatchi, S.; Smith, T.B.; Zutta, B.R.; Chaves, J. A.; Milá, B.; Graham, C. H. Predicting species distributions across the Amazonian and Andean regions using remote sensing data. *J. Biogeogr.* **2008**, 35, 7, 1160-1176. doi: 10.1111/j.1365-2699.2007.01858.x

Phillips, S.J.; Anderson, R.P.; Schapire, R.E. Maximum entropy modeling of species geographic distributions. *Ecol. Model.* **2006**, 190, 231-259, doi: 10.1016/j.ecolmodel.2005.03.026.

Phillips, S.J.; Dudík, M. Modeling of species distributions with Maxent: new extensions and a comprehensive evaluation. *Ecography* **2008**, 31, 161-175, doi: 10.1111/j.0906-7590.2008.5203.x.

Warren, D.L.; Glor, R.E.; Turelli, M. Environmental Niche Equivalency Versus Conservatism: Quantitative Approaches to Niche Evolution. *Evolution* **2008**, 62, 2868-2883, doi: 10.1111/j.1558-5646.2008.00482.x.

Warren, D.L.; Glor, R.E.; Turelli, M. ENMTools: a toolbox for comparative studies of environmental niche models. *Ecography* **2010**, 33, 607-611, doi: 10.1111/j.1600-0587.2009.06142.x.
